# Chemotherapy-related cardiotoxicity and its symptoms in patients with breast cancer: a scoping review

**DOI:** 10.1186/s13643-024-02588-z

**Published:** 2024-06-27

**Authors:** Hyunjoo Kim, Bomi Hong, Sanghee Kim, Seok-Min Kang, Jeongok Park

**Affiliations:** 1 Graduate School, College of Nursing, Yonsei University, 50 Yonsei-ro, Seoul, South Korea; 2 Severance Cardiovascular Hospital, Yonsei University, 50-1 Yonsei-ro, Seoul, South Korea; 3 College of Nursing and Brain Korea 21 FOUR Project, Yonsei University, 50-1 Yonsei-ro, Seoul, South Korea; 4 College of Nursing and Mo-Im Kim Nursing Research Institute, Yonsei University, 50-1 Yonsei-ro, Seoul, South Korea; 5 Department of Internal Medicine, Division of Cardiology, Heart Failure Center, Severance Hospital, Yonsei University, 50-1 Yonsei-ro, Seoul, South Korea

**Keywords:** Breast cancer, Chemotherapy, Cardiotoxicity, Symptom

## Abstract

**Background:**

Chemotherapy-related cardiotoxicity is a significant concern because it is a major cause of morbidity. This study aimed to provide in-depth information on the symptoms of chemotherapy-related cardiotoxicity (CRCT) by exploring literature that concurrently reports the types and symptoms of CRCT in patients with breast cancer.

**Methods:**

A scoping review was performed according to an a priori protocol using the Joanna Briggs Institute’s guidelines. The participants were patients with breast cancer. The concept was the literature of specifically reported symptoms directly matched with CRCT and the literature, in English, from 2010, and the context was open. The search strategy included four keywords: “breast cancer,” “chemotherapy,” “cardiotoxicity,” and “symptoms.” All types of research designs were included; however, studies involving patients with other cancer types, animal subjects, and symptoms not directly related to CRCT were excluded. Data were extracted and presented including tables and figures.

**Results:**

A total of 29 articles were included in the study, consisting of 23 case reports, 4 retrospective studies, and 2 prospective studies. There were no restrictions on the participants’ sex; however, all of them were women, except for one case report. The most used chemotherapy regimens were trastuzumab, capecitabine, and doxorubicin or epirubicin. The primary CRCT identified were myocardial dysfunction and heart failure, followed by coronary artery disease, pulmonary hypertension, and other conditions. Major tests used to diagnose CRCT include echocardiography, electrocardiography, serum cardiac enzymes, coronary angiography, computed tomography, and magnetic resonance imaging. In all case reports, CRCT was diagnosed through an incidental checkup according to the patient’s symptom presentation; however, only 10 of these studies showed a baseline checkup before chemotherapy. The five most common CRCT symptoms were dyspnea, chest pain, peripheral edema, fatigue, and palpitations, which were assessed by patient-reported symptom presentation rather than using a symptom assessment tool. Dyspnea with trastuzumab treatment and chest pain with capecitabine treatment were particularly characteristic. The time for first symptom onset after chemotherapy ranged from 1 hour to 300 days, with anthracycline-based regimens requiring 3–55 days, trastuzumab requiring 60–300 days, and capecitabine requiring 1–7 days.

**Conclusions:**

This scoping review allowed data mapping according to the study design and chemotherapy regimens. Cardiac assessments for CRCT diagnosis were performed according to the patient’s symptoms. There were approximately five types of typical CRCT symptoms, and the timing of symptom occurrence varied. Therefore, developing and applying a CRCT-specific and user-friendly symptom assessment tool are expected to help healthcare providers and patients manage CRCT symptoms effectively.

**Supplementary Information:**

The online version contains supplementary material available at 10.1186/s13643-024-02588-z.

## Background

Breast cancer is currently the most common cancer worldwide. Its incidence and mortality rates in East Asia in 2020 accounted for 24% and 20% of the global rates, respectively, and these rates are expected to continue increasing until 2040 [1]. In the USA, since the mid-2000s, the incidence rate of breast cancer has been increasing by 0.5% annually, while the mortality rate has been decreasing by 1% per year from 2011 to 2020 [2]. Despite the improved long-term survival rate in patients with breast cancer due to the development of chemotherapy, the literature has highlighted that cardiotoxicity, a cardiac problem caused by chemotherapy, could be a significant cause of death among these patients [3]. Chemotherapy-related cardiotoxicity (CRCT) can interfere with cancer treatment and progress to congestive heart failure during or after chemotherapy [4], potentially lowering the survival rate and quality of life of patients with cancer [5].

The term cardiotoxicity was first used in the 1970s to describe cardiac complications resulting from chemotherapy regimens, such as anthracyclines and 5-fluorouracil. The early definition of cardiotoxicity centered around heart failure, but the current definition is broad and still imprecise [6]. The 2022 guidelines on cardio-oncology from the European Society of Cardiology (ESC) define cardiotoxicity as including cardiac dysfunction, myocarditis, vascular toxicity, arterial hypertension, and cardiac arrhythmias. Some of these definitions reflect the symptoms. For example, cardiac dysfunction, which accounts for 48% of cardiotoxicity in patients with cancer, is divided into asymptomatic and symptomatic cardiac dysfunction. Asymptomatic cardiac dysfunction is defined based on left ventricular ejection fraction (LVEF), myocardial global longitudinal strain, and cardiac biomarkers. Symptomatic cardiac dysfunction indicates heart failure and presents with ankle swelling, breathlessness, and fatigue [7]. The ESC guidelines for heart failure present more than 20 types of symptoms [8]; however, to the best of our knowledge, few studies have been conducted to determine which heart failure symptoms and their characteristics are associated with CRCT in patients with breast cancer. Similarly, there is a lack of information related to vascular toxicity such as myocardial infarction [7].

Professional societies in cardiology and oncology have proposed guidelines for the prevention and management of cardiotoxicity in patients with cancer. According to the American Society of Clinical Oncology and the ESC, it is recommended to identify high-risk patients, comprehensively evaluate clinical signs and symptoms associated with CRCT, and conduct cardiac evaluations before, during, and after chemotherapy [7, 9, 10]. In addition, guidelines for patients with cancer, including those for breast cancer survivorship care, emphasize that patients should be aware of the potential risk of CRCT and report symptoms, such as fatigue or shortness of breath to their healthcare providers [7, 11, 12]. Although these guidelines encompass cardiac monitoring as well as symptom observation, many studies have focused solely on objective diagnostic tests, such as echocardiography, cardiac magnetic resonance, and cardiac biomarkers [13–22], which means that there is little interest in CRCT symptoms in patients under breast cancer care.

This lack of interest in CRCT symptoms may be related to the absence of a specific symptom assessment tool for CRCT. Symptom monitoring of CRCT in patients with breast cancer was conducted through patient interviews and reported using the appropriate terminology [23]. In terms of interviews, patients with cancer experienced the burden of expressing symptoms between cardiovascular problems and cancer treatment. Qualitative research on patients with cancer indicates that these patients experience a daily battle to distinguish the symptoms they experience during chemotherapy [24]. To reduce the burden of identifying CRCT symptoms, it is crucial to educate patients with breast cancer undergoing chemotherapy about these symptoms. To report cardiotoxicity, healthcare providers in oncology can use a dictionary of terms called the Common Terminology Criteria for Adverse Events (CTCAE) for reporting adverse events in patients with cancer [25]. Patients can also use Patient-Reported Outcome (PRO), which allows unfiltered reporting of symptoms directly to the clinical database [26]. PRO consists of 78 symptomatic adverse events out of approximately 1,000 types of CTCAE [27]. Basch et al. suggested that PRO could enable healthcare providers to identify patient symptoms before they worsen, thereby improving the overall survival rate of patients with metastatic cancer [28]. This finding implies that symptoms can provide valuable clues for enhancing the timeliness and accuracy of clinical assessments of CRCT [29]. Therefore, it is necessary to explore the scope of research focusing on CRCT symptoms for prevention and early detection of CRCT in patients with breast cancer. The detailed research questions are as follows:

1. What are the general characteristics of the studies related to CRCT in patients with breast cancer?
2. What diagnostic tools and monitoring practices are used to detect CRCT?
3. What are the characteristics and progression of symptoms associated with CRCT?

## Methods

A scoping review is a research method for synthesizing evidence that involves mapping the scope of evidence on a particular topic [30]. It aims to clarify key concepts and definitions, identify key characteristics of factors related to a concept, and highlight gaps or areas for further research [30]. This study used a scoping review methodology based on the Joanna Briggs Institute (JBI) framework. The JBI methodology, refined from the framework initially developed by Arksey and O’Malley [31], involves developing a research question, establishing detailed inclusion and exclusion criteria, and selecting and analyzing literature accordingly [32]. In contrast to systematic reviews, scoping reviews can encompass a variety of study designs and are particularly suitable when the topic has not been extensively studied [33]; hence, the decision was made to conduct a scoping review.

### Development of a scoping review protocol

To conduct this review, an a priori scoping review protocol was developed to enhance transparency and increase the usefulness and reliability of the results. The protocol included the title, objective, review questions, introduction, eligibility criteria, participants, concept, context, types of evidence source, methods, search strategy, source of evidence selection, data extraction, data analysis and presentation, and deviation from the protocol [34] (Supplementary File 1).

### Eligibility criteria

A participant-concept-context (PCC) framework was constructed based on the following research criteria. The participants were patients with breast cancer. The concept was that studies that specifically reported symptoms directly matched to CRCT in patients with breast cancer and the literature, published in English since 2010, in line with the year the CRCT guidelines were announced by the Cardio-Oncology Society. The context was open. We included all types of research designs. The exclusion criteria were studies that included patients with other types of cancer, involved animal subjects, and reported symptoms not directly related to CRCT.

### Search strategy

The keywords consisted of “breast cancer,” “chemotherapy,” “cardiotoxicity,” and “symptoms.” The keywords for “cardiotoxicity” were constructed according to the clinical cardiotoxicity report and ESC guidelines [7, 35]. The keywords for “symptoms” included 40 specific symptoms of arrhythmia, heart failure, and cardiac problems [36, 37] (Supplementary Table 1). We used PubMed, Embase, and CINAHL.

### Source of evidence selection

Duplicate studies were removed using EndNote 21. The titles and abstracts were then reviewed according to the inclusion criteria, the primary literature was selected, and the final literature was selected through a full-text review. Any disagreements were resolved through discussions between the investigators.

### Data extraction

The data from the literature included the general characteristics of the study, as well as information on the patients, chemotherapy, cardiotoxicity, and symptoms. The general characteristics of the study included author, publication year, country of origin, study design; patient information including sample size, sex, age, cancer type, and cancer stage; chemotherapy information including chemotherapy regimen; cardiotoxicity information including type of cardiotoxicity, diagnostic tests, and times of assessment; and symptom information including type of symptom, characteristics of symptom worsening or improvement, onset time, progression time, and time to symptom improvement. Information on whether to receive chemotherapy after the diagnosis of cardiotoxicity was explored.

### Data analysis and presentation

The contents of the included studies were divided into three categories: (1) general characteristics, which encompassed study designs, patients, and medications; (2) type of CRCT and cardiac assessment for CRCT; and (3) characteristics and progression of the symptoms associated with CRCT. CRCT symptom-related data are presented in tables and figures.

## Results

**Fig. 1:**
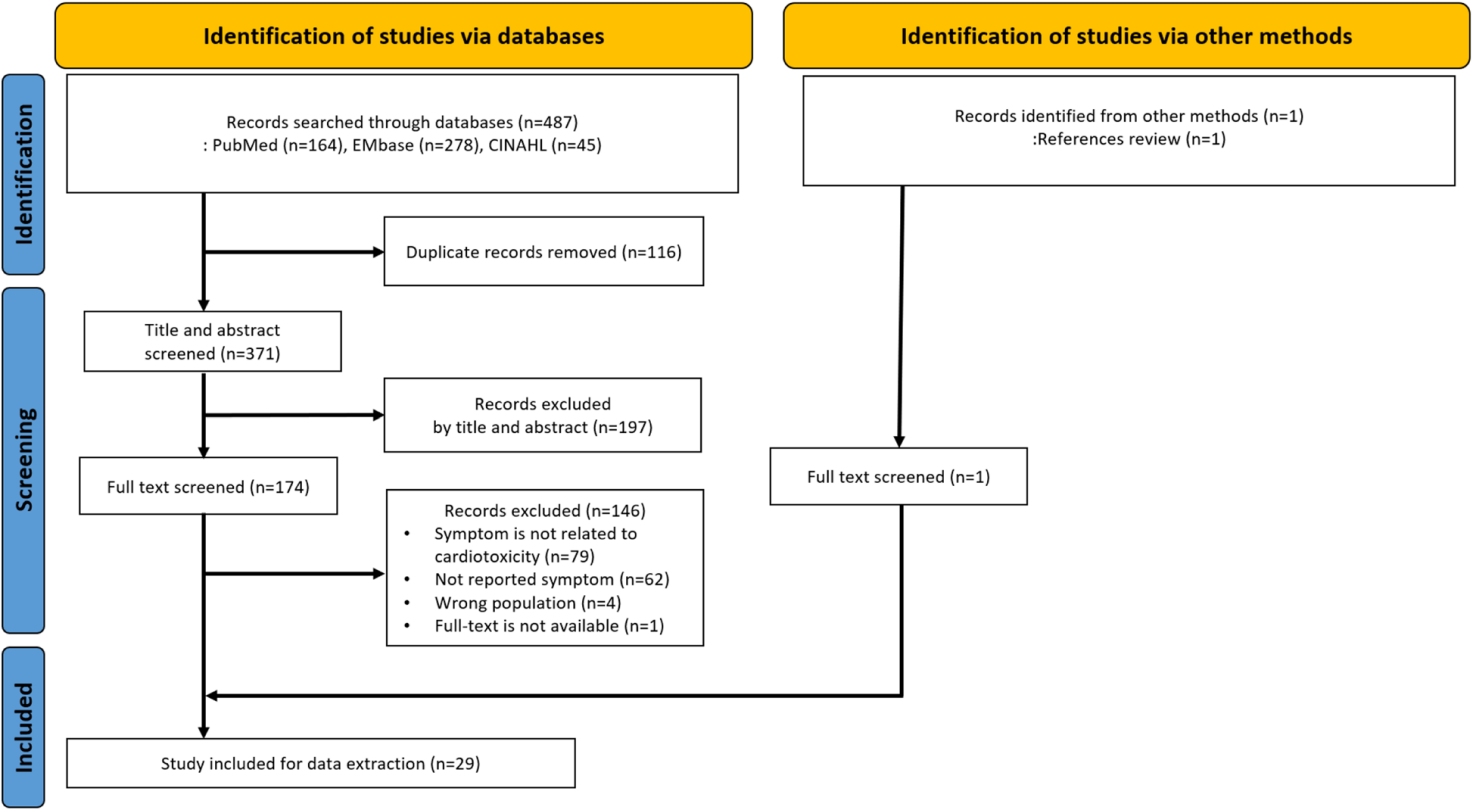
Preferred reporting items for systematic reviews flowchart

In total, 487 studies were identified through database searches, and 116 duplicates were subsequently removed. After reviewing the titles and abstracts, we excluded 197 studies in which participants had cancers other than breast cancer, no symptoms, or symptom-related expressions. Of the remaining 174 studies, 146 were excluded after full-text review. Among the excluded studies, 79 were mainly clinical trials that the symptoms were not directly related to CRCT, 62 did not report specific symptoms, four were in the wrong population, and one was unavailable for full-text review. An additional study was included after a review of references, bringing the final count to 29 studies included in the analysis (Fig. 1).

### General characteristics of studies including designs, sex and age, chemotherapy regimen, and CRCT criteria

Table 1 presents the general characteristics of the studies included in this review. The majority of these studies were published in the USA (*n*=14), with Japan (*n*=3), and Romania (*n*=2) following. The study designs primarily consisted of case reports (*n*=23), retrospective studies (*n*=4), and prospective studies (*n*=2).

**Table 1:** General characteristics of the studies (*n*=29)

**First authors, years**	**Country**	**Sample size** **sex/age**	**Cancer stage**	**Chemotherapy**	**Previous treatment**	**Type or criteria of chemotherapy-related cardiotoxicity**
▪ Prospective studies						
Bendahou, 2022^a^	Morocco	795 Sex: ND Age: 53±11	Not limited	Trastuzumab	Not described	Decrease in LVEF over 10% at the lower limit of normal of 50%
Chang, 2016	Taiwan	35 Sex: ND Age: 45.33±8.48	Not limited	Epirubicin	Epirubicin	Heart failure (by impaired RVLS-FW, LVGLS, or LVEF)
▪ Retrospective studies						
Aldiab, 2010 [38]	Saudi Arabia	98 Sex: ND Age: 20–80	Not limited	Adjuvant trastuzumab	Doxorubicin	Heart failure (LVEF drops by 10% of the original value or below the normal value)
Russell, 2010 [39]	United States	173 Sex: ND Age: ND	Not limited	Adjuvant doxorubicin, cyclophosphamide, and paclitaxel with or without trastuzumab	Not described	Heart failure (LVEF drops by 10% of the original value or below 50%, signs, and symptoms of CHF)
Masson, 2013^a^	France	155 Sex: ND Age: 52.9 in group 1, 54.4 in group 2	Not limited	Trastuzumab	Not described	10% decrease of LVEF or/and lower than 50%
Polk, 2016 [40]	Denmark	452 Sex: woman Age: 28–88	Advanced stage	Palliative capecitabine with or without trastuzumab	Chemotherapy (anthracycline, trastuzumab); Radiation therapy	Significant symptoms of likely cardiac origin EKG change
▪ Case studies						
Szmit, 2010	Poland	F/42	Stage 1	Adjuvant trastuzumab	Chemotherapy (doxorubicin 360mg/m^2^, herceptin 26mg/kg cyclophosphamide 900mg); Operation; Radiation therapy (left breast)	Acute heart failure with LV thrombus
Güvenç, 2012	Turkey	F/46	Stage 4	Palliative capecitabine	Operation; Hormone therapy (tamoxifen)	STEMI, Acute coronary syndrome
Santiago, 2013	United States	F/32	Stage 4	Palliative ixabepilone	Chemotherapy (doxorubicin, cyclophosphamide, paclitaxel); Radiation therapy (right breast); Operation	EKG change, R/O cardiogenic shock or heart failure
Beceanu, 2015^a^	Romania	F/39	Stage 4	Trastuzumab	Chemotherapy (docetaxel, trastuzumab)	Heart failure (cardiomyopathy)
Guta, 2016^a^	Romania	F/65	Not described	Cyclophosphamide	Chemotherapy, Radiation therapy, Operation	Heart failure (atrial fibrillation)
Henry, 2016	United States	F/41	Stage 4	Palliative lapatinib and capecitabine	Chemotherapy (trastuzumab, pertuzumab, docetaxel, trastuzumab emtansine); Hormone therapy (tamoxifen); Operation	Coronary vasospasm
Kwon, 2016	United States	F/43	Stage 4	Palliative trastuzumab emtansine (T-DM1)	Chemotherapy (5-fluorouracil, epirubicin, cyclophosphamide, paclitaxel, trastuzumab); Operation; Radiation	Pulmonary arterial hypertension
Inoue, 2017	Japan	F/55	Stage 4	Palliative epirubicin, cyclophosphamide	None	Periaortitis
Johnson, 2017	United States	M/66	Stage 2	Adjuvant taxol, tamoxifen, carboplatin	Operation	Acute coronary syndrome
Saeed, 2018^a^	United States	F/70	Stage 4	Capecitabine	Chemotherapy	Coronary vasospasm
Hartopo, 2020^a^	Indonesia	F/63	Not described	Taxane	Not described	Heart failure
Maria, 2020	India	F/63	Stage 3	Adjuvant doxorubicin, cyclophosphamide	Hormone therapy; Operation; Radiation therapy (left)	NSTEMI, acute coronary syndrome
Masson, 2020	United States	F/70	Not described	Adjuvant trastuzumab	Chemotherapy(paclitaxel); Operation	Acute heart failure with LBBB
Powers, 2020^a^	United States	F/65	Stage 4	Capecitabine	Not described	Coronary vasospasm, Takotsubo cardiomyopathy
Bhattacharya, 2021^a^	United States	F/84	Not applicable	Capecitabine	Not described	Takostubo cardiomyopathy
Oyakawa, 2021	Japan	F/68	Stage 4	Palliative abemaciclib, fulvestrant	Chemotherapy (paclitaxel, bevacizumab); Hormone therapy (letrozole); Operation	Heart failure (myocardial dysfunction)
Mazek, 2021^a^	United States	F/52	Stage4	Capecitabine	Not described	Coronary vasospasm
Conte, 2022^a^	Italy	F/50	Not described	Epirubicin and cyclophosphamide	None	Heart failure (LVEF 15%)
Javed, 2022	United States	F/59	Not described	Doxorubicin and cyclophosphamide	None	Pulmonary arterial hypertension
Muco, 2022 [41]	United States	F/32	Not described	Capecitabine	Chemotherapy (carboplatin, paclitaxel); Operation	Coronary vasospasm (ventricular fibrillation)
Ushiyama, 2023	Japan	F/46	Stage 2	Adjuvant trastuzumab, tamoxifen	Chemotherapy (anthracycline, taxane, trastuzumab); Operation	Acute myocarditis, cardiogenic arrest
Ahmad, 2023^a^	United States	F/49	Stage 4	Abemaciclib, letrozole	Not described	Heart failure (LVEF 43%)
Angelini, 2023	United States	F/86	Stage4	Vinorelbine, cisplatin, trastuzumab, pertuzumab, paclitaxel, and romiplostim	None	Coronary vasospasm, Takotsubo cardiomyopathy

*ND* Not described, *LVEF* Left ventricular ejection fraction, *RVLS-FW* Right ventricular longitudinal strain-free wall, *LVGLS* Left ventricular global longitudinal strain, *EKG* Electrocardiogram, *STEMI* ST Segment Elevation myocardial infarction, *NSTEMI* Non-ST segment elevation myocardial infarction, *R/O* Rule out

^a^Conference proceeding

All case reports involved female patients, except for one involving a male patient. Five quantitative studies did not specify or limit the sex of the participants, and one retrospective study included only female patients. In terms of cancer stage, the majority of studies involved patients with advanced breast cancer (*n*=13), while a smaller number involved patients with early-stage breast cancer (*n*=4). Twelve studies did not specify the cancer stage. Approximately 20 types of chemotherapy regimens are currently in use. Trastuzumab, which is a human epidermal growth factor receptor 2 (HER2) blocker, was mentioned in the majority of studies (*n*=8), followed by capecitabine (an antimetabolite) (*n*=7), and doxorubicin or epirubicin (anthracycline-based chemotherapy) (*n*=6). Current chemotherapy and previous treatment methods were described together, with the exception of eight studies. Six quantitative studies defined the CRCT criteria, five of which were based on decreased LVEF and one of which was based on significant cardiac symptoms and/or electrocardiogram changes. Twenty-three case reports described the cardiovascular diagnosis as CRCT.

### Diagnostic tools and monitoring practice for CRCT

Table 2 displays the types of CRCT, diagnostic tools, and times of cardiac assessment according to chemotherapy regimens. The most prevalent CRCT were myocardial dysfunction and heart failure, identified in 12 case studies, respectively. This was followed by coronary artery disease, represented in 8 case studies, pulmonary hypertension in 2 case studies, and a single case study of periaortitis. The most used test for diagnosing CRCT was echocardiography (*n*=22), followed by EKG (*n*=20), various types of cardiac enzymes (*n*=16), coronary angiography (CAG, *n*=12), computed tomography (*n*=6), and magnetic resonance imaging (MRI, *n*=4). Regarding the CRCT symptom assessment tools, the CTCAE was used in two studies, the New York Heart Association classification for heart failure in two studies, the dyspnea assessment scale in one study, and symptoms of cardiac origin, which consisted of chest pain, dyspnea, and palpitations in one study.

**Table 2:** Chemotherapy-related cardiotoxicities, diagnostic tools, and times of cardiac assessment according to chemotherapy regimens (*n*=29)

**First author, year**	**Administered chemotherapy**	**Type or criteria of chemotherapy-related cardiotoxicity**	**Diagnostic tool**	**Times of cardiac assessment**			
				**Regular checkup**			**Incidental checkup by symptom presentation**
				**Before treatment**	**During treatment**	**After treatment**	
▪** Anthracycline-based regimen**							
Maria, 2020	Doxorubicin and cyclophosphamide	NSTEMI, acute coronary syndrome	Echo, EKG, TMT, cardiac enzyme (CK, CK-MB, TnT), CAG	**√**			**√**
Javed, 2022	Doxorubicin and cyclophosphamide	Pulmonary arterial hypertension	Echo, CAG	**√**			**√**
Russell, 2010 [39]	Doxorubicin, cyclophosphamide, and paclitaxel with or without trastuzumab	Symptomatic heart failure	MUGA/Echo, chest X-ray, physical exam record, NYHA class, NCI-CTC 2.0	**√**	**√**	**√**	
Chang, 2016	Epirubicin	Heart failure (by impaired RVLS-FW, LVGLS, LVEF)	Echo, cardiac enzyme (BNP), dyspnea assessment scale	**√**	**√**		
Inoue, 2017	Epirubicin and cyclophosphamide	Periaortitis	CT				**√**
Conte, 2022	Epirubicin and cyclophosphamide	Heart failure (LVEF 15%)	Echo, EKG, CMR	**√**			**√**
▪** Human epidermal growth factor receptor 2 blockers**							
Szmit, 2010	Trastuzumab	Acute heart failure with LV thrombus	Echo, EKG, MRI, chest X-ray, cardiac enzyme (NT-proBNP)	**√**			**√**
Aldiab, 2010 [38]	Trastuzumab	Heart failure (EF drops by 10% of the original value or below the normal value)	MUGA or Echo, NYHA class	**√**	**√**		
Masson, 2013	Trastuzumab	10% decrease of LVEF or/and lower than 50%	Radionuclide ventriculography, heart failure symptoms	**√**	**√**		
Beceanu, 2015	Trastuzumab	Heart failure (cardiomyopathy)	Echo	√	√		√
Masson, 2020	Trastuzumab	Acute heart failure with LBBB	Echo, EKG, cardiac enzyme (troponin), CT, MRI, CAG	**√**	**√**		**√**
Bendahou, 2022	Trastuzumab	Heart failure: 107(13.4%) (decrease in LVEF over 10% at the lower limit of normal of 50%)	Echo, NYHA class	**√**	**√**	√	
Ushiyama, 2023	Trastuzumab, tamoxifen	Acute myocarditis, cardiac arrest	EKG, cardiac enzyme (CK, CK-MB, TnT, proBNP), CAG, myocardial biopsy	**√**			**√**
Kwon, 2016	Trastuzumab emtansine	Pulmonary arterial hypertension	Echo, CT, ventilation perfusion scan, PFT, CAG, right-sided catheterization				**√**
▪** Antimetabolites**							
Güvenç, 2012	Capecitabine	STEMI, Acute coronary syndrome	CAG, EKG, cardiac enzyme				**√**
Saeed, 2018	Capecitabine	Coronary vasospasm	EKG, cardiac enzyme (CK, CK-MB), Echo, MRI				**√**
Powers, 2020	Capecitabine	Coronary vasospasm, Takotsubo cardiomyopathy	EKG, cardiac enzyme(troponin), Echo, CAG				**√**
Mazek, 2021	Capecitabine	Coronary vasospasm	EKG, cardiac enzyme (troponin), CAG				**√**
Muco, 2022	Capecitabine	Coronary vasospasm (ventricular fibrillation)	CT, Echo, EKG, cardiac enzyme, CAG				**√**
Henry, 2016	Capecitabine and lapatinib	Coronary vasospasm	CT, Echo, EKG, cardiac enzyme (troponin)				**√**
Polk, 2016 [40]	Capecitabine with or without trastuzumab	Symptoms of likely cardiac origin, EKG change	NCI CTC v3.0, EKG, cardiologist’s review	**√**			**√**
▪** Taxol-based regimen**							
Johnson, 2017	Taxol, tamoxifen, carboplatin	Acute coronary syndrome	CAG, EKG, Echo, cardiac enzyme (TnI)				**√**
Hartopo, 2020	Taxane	Heart failure	EKG, Echo	**√**			**√**
Bhattacharya, 2021	Docetaxel, cyclophosphamide	Takostubo cardiomyopathy	EKG, Echo, CAG, left heart catheterization				**√**
▪** Abemaciclib based regimen**							
Oyakawa, 2021	Abemciclib, fulvestrant	Heart failure (myocardial dysfunction)	EKG, Echo, cardiac enzyme (BNP, TnI), chest X-ray, MRI	**√**			**√**
Ahmad, 2023	Abemaciclib, letrozole	Heart failure (LVEF 43%)	EKG, Echo, CMR, chest X-ray				**√**
▪** Others**							
Santiago, 2013	Ixabepilone	EKG change, R/O acute heart failure with cardiogenic shock	CT, EKG, cardiac enzyme (TnI, BNP)	**√**			**√**
Guta, 2016	Cyclophosphamide	Heart failure (A-fib)	Echo, EKG, cardiac enzyme(proBNP)				**√**
Angelini, 2023	Vinorelbine, cisplatin, trastuzumab, pertuzumab, paclitaxel, and romiplostim	Coronary vasospasm, Takotsubo cardiomyopathy	EKG, Echo, cardiac enzyme (high-sensitivity troponin, BNP), CAG				**√**

*NSTEMI* Non-ST segment elevation myocardial infarction, *Echo* Echocardiography, *EKG* Electrocardiogram, *CK* Creatine kinase, *CK-MB* Creatine kinase-myocardial band, *TnT* Troponin T, *TMT* Treadmill test, *CAG* Coronary angiography, *MUGA* Multigated acquisition scan, *NYHA* New York Heart Association, *NCI-CTC* National Cancer Institute-Common toxicity criteria, *RVLS-FW* Right ventricular longitudinal strain_free wall, *LVGLS* Left ventricular global longitudinal strain, *LVEF* Left ventricular ejection fraction, *BNP* Brain natriuretic peptide, *CT* Computed tomography, *CMR* Cardiac magnetic resonance imaging, *MRI* : Magnetic Resonance Imaging, *PFT* Pulmonary function test, *STEMI* ST elevated myocardial infarction, *TnI* Troponin I

Regarding the times of cardiac evaluation, two studies performed regular cardiac checkups including before, during, and after chemotherapy. There were 10 case studies and six quantitative studies describing cardiac function testing before chemotherapy, of which seven studies performed regular cardiac screening tests and two studies mentioned cardiac screening even after the completion of chemotherapy. The frequency of regular checkups varied from every 3 months to every two to four cycles. In all case reports (*n*=23), CRCT were diagnosed through incidental checkups based on patients’ symptom presentation, and in most cases, several tests were performed subsequentially for CRCT diagnosis. In one case study, cardiac evaluation was conducted 3 days after the patient’s initial symptom presentation, when the symptoms became more severe.

### Characteristics and progression of symptoms associated with CRCT

Table 3 shows the descriptive scope of the CRCT-related symptoms according to the chemotherapy regimens used in the included studies. The mapping factors included initial symptoms, symptom onset or severity, symptom progression, medical management, and CRCT results. One of the most frequent symptoms associated with CRCT was dyspnea, which was discussed in 19 studies and described as difficulty in breathing, shortness of breath, or New York Heart Association (NYHA) class II or III. When dyspnea appeared as the initial symptom of CRCT, the symptom progression was worsening in eight case studies and persistent in two cases. Chest pain was described in 12 studies as a symptom characterized by a squeezing, tingling, burning, tightened, or atypical feeling that was relieved by rest and exacerbated by exertion. Other symptoms included peripheral edema (*n*=6), fatigue (*n*=5), and palpitation (*n*=2). The symptoms were assessed by patient-reported symptom presentation rather than using a symptom assessment tool.

**Table 3:** The symptom assessment and management of chemotherapy-related cardiotoxicity (*n*=29)

**First author, year**	**Type or criteria of chemotherapy-related cardiotoxicity**	**Administered chemotherapy**	**Initial symptom**	**Symptom onset/severity**	**Symptom progression**	**Management**	**Results of chemotherapy-related cardiotoxicity**
▪** Anthracycline-based regimen**							
Maria, 2020	NSTEMI, acute coronary syndrome	Doxorubicin and cyclophosphamide	Chest discomfort and chest pain, atypical	55 days/ND	ND	Regimen changed after cardiac medication	Improved after cardiac medication
Javed, 2022	Pulmonary arterial hypertension	Doxorubicin and cyclophosphamide	Lower extremity edema, shortness of breath	7 days/ND	Worsening	Regimen stop and change into other	Improved in 5 months
Russell, 2010	Symptomatic heart failure	Doxorubicin, cyclophosphamide, and paclitaxel with or without trastuzumab	Heart failure symptoms: dyspnea, orthopnea, shortness of breath, exertional dyspnea, pedal edema, weight gain	ND/NYHA class I~IV, NCI-CTC 3~5	ND	Cardiac medication	Complete or partial recovery in 42.9% of CTx alone group and 86.1% of CTx with trastuzumab group
Chang, 2016	Heart failure	Epirubicin	Dyspnea on exertion	ND/ND	ND	ND	ND
Inoue, 2017	Periaortitis	Epirubicin and cyclophosphamide	Fever, stomatitis	11 days/ND	Back pain	Epirubicin stop and antibiotics use	Symptom recovery in 2 weeks Discharged on the 33rd day of admission
Conte, 2022	Heart failure	Epirubicin and cyclophosphamide	Asthenia, vomiting, dyspnea	3 days/ND	Persistent for 3 days	Cardiac medication	ND
▪** Human epidermal growth factor receptor 2 blockers**							
Szmit, 2010	Heart failure	Trastuzumab	Dyspnea, leg edema	90 days/NYHA class II	Worsening for 3 weeks	Trastuzumab stop and cardiac medication	Complete recovery in 4 months after diagnosis
Masson, 2020	Heart failure	Trastuzumab	Shortness of breath, cough, chest pain	180 days/ND	Respiratory failure after ER arrival (influenza A, H1N1 virus)	Trastuzumab stop, referred to cardiologist, with or without cardiac medications	Recovered LVEF 4 weeks after discharge with intermittent left bundle branch block for one year
Aldiab, 2010 [38]	Decreased LVEF	Trastuzumab	Dyspnea on exertion	ND/NYHA class I, II, III	ND	Trastuzumab stop or therapeutic break	Complete or partial recovery
Masson, 2013	Heart failure	Trastuzumab	Dyspnea, orthopnea, edema	ND/ND	ND	Trastuzumab stop and cardiac medication	ND
Beceanu, 2015	Heart failure	Trastuzumab	Shortness of breath, orthopnea, thoracic pain	300 days/NYHA class II	Progressive worsening	Trastuzumab stop, intubation, ICU care, wearable defibrillator with cardiac medication	Asymptomatic after 2 months (not improved LVEF: 43% at the 3rd month)
Bendahou, 2022	Heart failure	Trastuzumab	Dyspnea	ND/NYHA class II to III	ND	Trastuzumab stop with cardiac medication	Recovery: 9 patients in 6 months
Kwon, 2016	Acute myocarditis	T-DM1 with/without pertuzumab	Dyspnea on exertion, fatigue, hereditary hemorrhagic telangiectasia, with acne-like rash on chest	106 days/ND	Progressive worsening of dyspnea on exertion for several months	Trastuzumab stop, ICU care, intubation, IABP, ECMO, Steroid/immunoglobulin therapy, PM insertion, cardiac medication	Improved after cardiac medication.
Ushiyama, 2023	Pulmonary arterial hypertension	Trastuzumab, tamoxifen	Dyspnea, fever	60 days/ND	Worsening condition: cardiac arrest	T-DM1stop and cardiac medication	Symptom improved in 14 days, discharged on the 28th day with PM
▪** Antimetabolites**							
Güvenç, 2012	STEMI, Acute coronary syndrome	Capecitabine	Chest pain, squeezing	1 day/ND	Persistent for 2 hours	Capecitabine stop and coronary intervention	Improved and discharged on the 7th day of admission
Saeed, 2018	Coronary vasospasm	Capecitabine	Chest pain and fatigue	7 days/ND	Intermittent	Capecitabine dose reduction with cardiac medication	Improved after cardiac medication
Powers, 2020	Coronary vasospasm, Takotsubo cardiomyopathy	Capecitabine	Chest pain, severe	ND/ND	Persistent	Capecitabine stop and cardiac medication	Improved in 1 week (normalized LVEF)
Mazek, 2021	Coronary vasospasm	Capecitabine	Chest pain and substernal chest tightness with diaphoresis, relieved by NTG	3 days/ND	Multiple episodes	Capecitabine stop, cardiac medication	Improved after cardiac medication
Muco, 2022	Coronary vasospasm (Ventricular fibrillation)	Capecitabine	Chest pain, burning substernal	1 day/ND	PEA cardiac arrest, anoxic brain injury, irreversible	Capecitabine stop and cardiac management	Discharged to a long-term care facility
Henry, 2016	Coronary vasospasm	Capecitabine	Chest pain, relieved by rest and exacerbated by exertion	3 days/ND	Persistent for 1 day	Capecitabine stop and aspirin and analgesics administration (refused angiography due to symptom improvement)	Improved after aspirin and analgesics administration
Polk, 2016 [40]	Symptoms of likely cardiac origin, EKG change	Capecitabine	Dyspnea, palpitation, and chest pain, oppressive, compressive, or radiating to another site	ND/ND	ND	Cardiac medication (45.4%), capecitabine stop (80%)	Not sufficient data
▪** Taxol-based regimen**							
Johnson, 2017	Acute coronary syndrome	Taxol, tamoxifen, carboplatin	Chest pain and nausea, with left arm tingling sense	126 days/ND	Persistent for 12 hours	Not described about further chemotherapy, coronary intervention and cardiac medication	Improved after coronary intervention and discharged home 2 days later
Hartopo, 2020	Heart failure	Taxane	Palpitation	22 days/ND	ND	Taxane stop, cardiac medication	Improved in 2 months (LVEF 46% to 60%)
Bhattacharya, 2021	Takostubo cardiomyopathy	Docetaxel, cyclophosphamide	Chest pain, left-sided crescendo-like, and dyspnea	7 days/ND	ND	ND	ND
▪ **Abemaciclib based regimen**							
Oyakawa, 2021	Heart failure	Abemaciclib	Breathlessness, edema of upper and lower extremities, fatigue, and weight gain	84 days/NYHA class II	ND	Abemaciclib stop and cardiac medication	Improved within 3 weeks under cardiac medication, normalized myocardial dysfunction after 2 months
Ahmad, 2023	Heart failure	Abemaciclib, letrozole	Dyspnea, peripheral edema	180 days/Not described	Persistent over 6months	ND	Improved after cardiac medication
▪** Others**							
Santiago, 2013	Heart failure	Ixabepilone	Dyspnea, abdominal pain	1 hour/ND	Worsening condition: cardiac decompensation and shock	Ixabepilone stop and ACLS	Death within a day
Guta, 2016	Heart failure	Cyclophosphamide	Dyspnea, fatigability	ND/ND	Worsening for 2 months	Cardiac medication	Symptom recovery after medication, Discharged on the 7^th^ day (LVEF 45%)
Angelini, 2023	Coronary vasospasm, Takotsubo cardiomyopathy	Vinorelbine, cisplatin, trastuzumab, pertuzumab, paclitaxel, and romiplostim	Chest pain, sudden onset and severe, dyspnea	7 days/ND	Resolved chest pain after NTG in 30min	CTx stop, PTCA with stent	Improved LVEF in 44 hours (LVEF 25% to 60%)^a^

*NSTEMI* Non-ST elevated myocardial infarction, *ND* Not described, *CTx* Chemotherapy, *LVEF* Left ventricular ejection fraction, *ER* Emergency room, *NYHA* New York Heart Association, *TDM-1* Trastuzumab emtansine, *ICU* Intensive care unit, *IABP* Intra-aortic balloon pump, *ECMO* Extracorporeal membrane oxygenation, *PM* Pacemaker, *STEMI* ST Elevated myocardial infarction, *NTG* Nitroglycerin, *PEA* Pulseless electrical activity, *EKG* Electrocardiogram, *ACLS* Advanced cardiovascular life support, *LBBB* Left bundle branch block, *PTCA* Percutaneous transluminal coronary angioplasty

^a^Died after 2days with cachexia and exhaustion

**Fig. 2:**
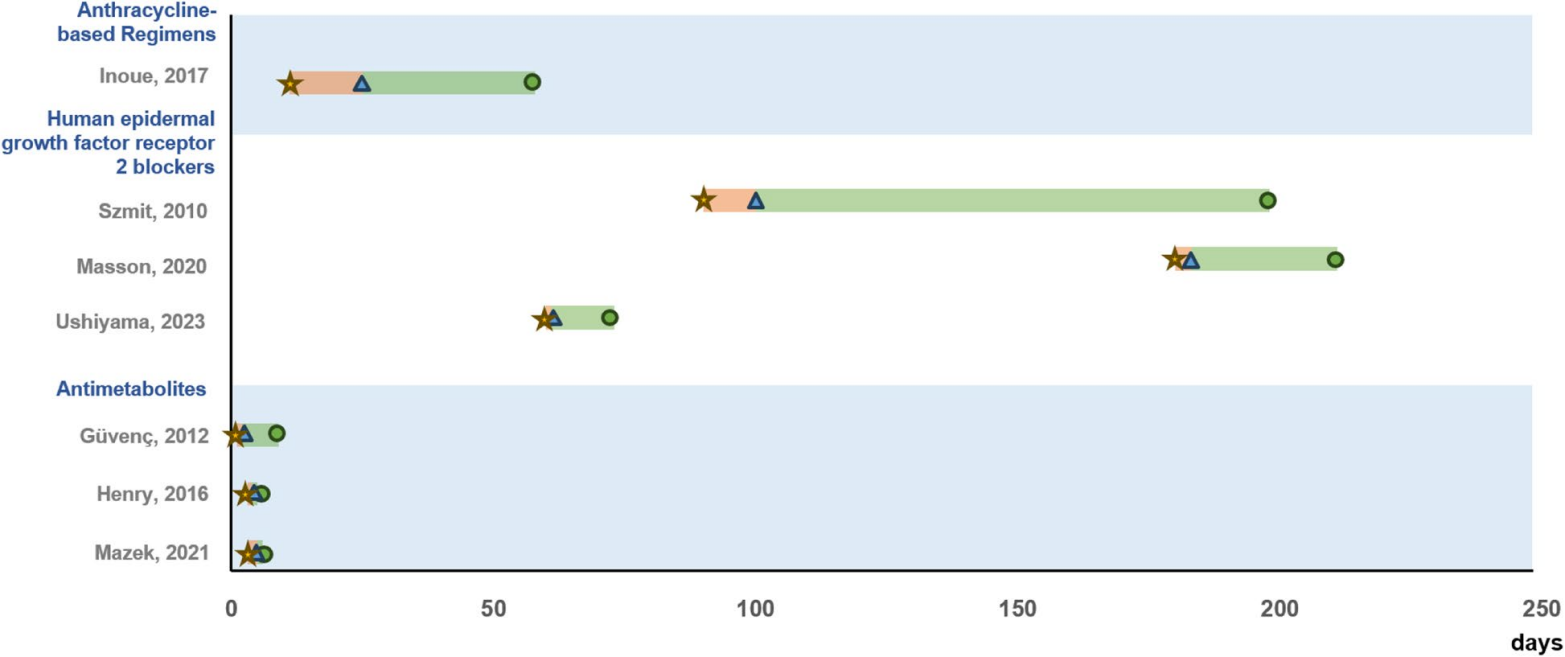
Symptom progression by chemotherapy regimens in case studies (*n*=7). Note.  The yellow star symbol means the date of symptom onset.  The blue triangle symbol means the date of diagnosis of CRCT. The green circle  symbol means the date of treatment finished.  The red line represents the period from symptom onset to diagnosis of CRCT.  The green line represents the period from symptom onset to the finished treatment of the symptom

The symptoms could be categorized based on the type of chemotherapy regimens used. In the case studies involving anthracycline-based regimen and HER2 blockers, dyspnea was the most frequently observed symptom (*n*=7), followed by peripheral edema (*n*=2), and chest pain or discomfort (*n*=2). In case studies where antimetabolites were used, specifically capecitabine, chest pain was a common and prominent symptom. This chest pain typically manifested between 1 and 7 days after drug administration and persisted until treatment. Notably, four out of seven patients reported this symptom on the first day of chemotherapy, according to the case reports. The time for first symptom onset after chemotherapy ranged from 1 hour to 300 days, with anthracycline-based regimens requiring 3–55 days, trastuzumab requiring 60–300 days, and capecitabine requiring 1–7 days. Figure 2 shows the progression of symptoms in case studies, detailing the time of symptom onset, the date of symptom reporting, and the date of treatment completion following the use of chemotherapy. The studies that did not specify any of the dates of symptom onset, reporting, and completion of treatment were excluded from the figure.

**Fig. 3:**
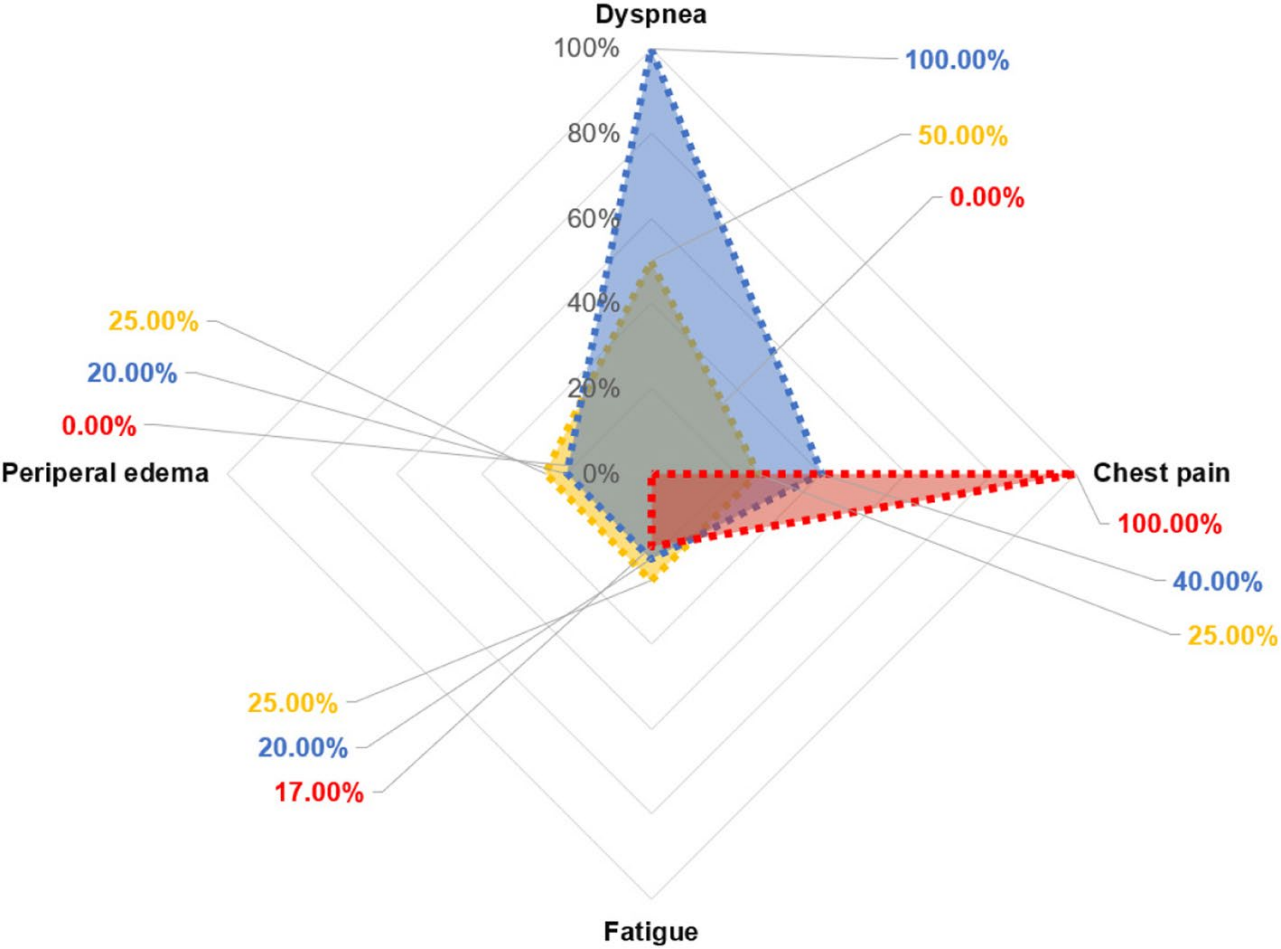
Proportion of symptoms according to major chemotherapy regimens in the studies (n=15). *Note.*  In the case studies using an Anthracycline-based regimen (*n*= 4), dyspnea, chest pain, peripheral edema, and fatigue were reported in 2 cases, 1 case, 1 case, and 1 case, respectively.  In the case studies using human epidermal growth factor receptor 2 blockers (*n*=5), dyspnea, chest pain, peripheral edema, and fatigue were reported in 5 cases, 2 case, 1 case, and 1 case, respectively.  In the case studies using antimetabolites (*n*=6), dyspnea, chest pain, peripheral edema, and fatigue were reported in 0 case, 6 cases, 0 case, and 1 case, respectively. Duplicates are present in studies

Figure 3 shows symptoms according to the main types of chemotherapy regimens reported in case studies. Dyspnea with trastuzumab and chest pain with capecitabine are particularly characteristic. A retrospective study included in this scoping review reported that chest pain was the most common symptom associated with capecitabine, followed by dyspnea and palpitation [40]. Furthermore, peripheral edema was primarily observed with anthracycline, alkylating, and HER2 blockers, while fatigue was noted with various anticancer drugs, irrespective of the type of chemotherapy regimen.

Ongoing chemotherapy was discontinued after CRCT was detected in 20 case studies. When patients presented symptoms indicative of CRCT, the majority were promptly hospitalized for further evaluation, medication, or interventional treatment. The majority of studies indicated the initiation of cardiac medication (*n*=21), with three case studies involving coronary intervention and two involving treatment with wearable devices. Most management procedures were conducted in a general ward or an intensive care unit.

In most case studies, symptoms improved following cardiac treatment, with either complete or partial recovery of LVEF observed in 19 instances. However, a few studies reported a poor prognosis, including two instances of death. LVEF recovered in most patients within 6 months when treated with an anthracycline-based regimen and HER2 blockers (Fig. 2). A retrospective study reported that the rates of complete or partial recovery of CRCT following treatment with doxorubicin-based chemotherapy and trastuzumab were 42.9% and 86.1%, respectively [39]. Another retrospective study noted that the recovery time of CRCT when treated with HER2 blockers increased in correlation with the severity of the NYHA class, ranging from 8 to 80 weeks [38]. In the case of the antimetabolite capecitabine, all patients recovered within a day to a week, except one patient who did not recover.

## Discussion

This scoping review was conducted to explore the scope of studies focusing on CRCT symptoms, including the general characteristics of the studies, diagnostic tools, monitoring practices related to detecting CRCT, and the characteristics and progression of symptoms associated with CRCT. The primary findings of this review were as follows: (1) common symptoms related to CRCT and differences in symptoms according to the chemotherapy regimens used were identified; (2) the symptoms reported by the patient served as clues to suspect a specific type of CRCT; and (3) regular monitoring practices for CRCT prevention and detection were insufficient.

First, the current study identified common symptoms such as dyspnea, chest pain, peripheral edema, fatigue, and palpitation associated with CRCT, as well as variations in symptoms depending on the chemotherapy regimen used in patients with breast cancer. Among these symptoms, dyspnea, edema, and chest pain were frequently observed in patients receiving anthracycline-based and/or HER2 blocker drugs. These symptoms, which are associated with heart failure, appeared later compared to those observed with capecitabine, as depicted in Fig. 2. This may be due to the known impact of anthracycline-based and/or HER2 blocker regimens on cardiomyocytes and other cells, leading to myocardial damage [42]. Therefore, the symptoms are related to heart failure, potentially resulting from the impairment of ventricular filling or ejection in patients undergoing treatment with these regimens [43].

In a similar vein, Attin et al. (2022) documented the occurrence of symptoms such as lower extremity edema, chest pain, difficulty breathing, and fatigue before the diagnosis of CRCT in women undergoing breast cancer treatment. They conducted a retrospective and longitudinal investigation of the symptoms, signs, and cardiac tests of 15 patients who experienced CRCT, using their electronic medical records. In their study, cardiotoxicity was defined by an echocardiogram or MRI showing a decrease in LVEF of 5 to 10%, with a specialist’s confirmation note. They compared the number of symptom occurrences during the first half of the year with those during the second half of the year prior to the diagnosis of cardiotoxicity. Specifically, the frequency of lower-extremity edema significantly increased from three occurrences in the first half of the year to 17 occurrences in the second half of the year. The frequency of symptoms for dyspnea and chest pain also increased from 10 and 8 times, respectively, to 16 and 14 times in the second half of the year. While there was limited information on the doses or timing of chemotherapy, 87% of the patients received the same chemotherapy regimens, namely anthracyclines and/or HER2 blockers [44]. This suggests that the increase in symptom occurrence over time may be related to the accumulation of anthracycline and the duration of anti-HER2 therapy [45].

Salyer et al. (2019) conducted a study on the prevalent symptoms of heart failure and their clustering. They identified three symptom clusters: sickness behavior, gastrointestinal disturbance, and discomfort of illness. Notably, dyspnea, edema, and pain were grouped into the discomfort of illness cluster, which aligns with the symptoms we observed in patients treated with anthracyclines and/or HER2 blockers [46]. Therefore, it is crucial for patients undergoing treatment with anthracyclines and/or HER2 blockers to be vigilant for symptoms such as dyspnea, edema, or chest pain, as these are indicative of heart failure.

Chest pain caused by vasospasm was a predominant symptom in patients taking antimetabolite regimens such as oral capecitabine, and it manifested as the following types of cardiotoxicities: vasospasm-related arrhythmia, myocardial disease, and ischemia [47]. Vasospasm can be triggered by endothelial dysfunction, hypersensitive vascular smooth muscle, reactive oxidative stress, or chemotherapy regimens [48, 49]. According to previous studies, in patients using antimetabolite drugs such as 5-fluorouracil or capecitabine, chest pain was usually reported to occur from several hours to 72 hours after the first administration [47, 50–53]. To detect chemotherapy-related coronary vasospasm in the early stage, it is recommended to carefully monitor typical or atypical symptoms of chest pain and EKG monitoring during drug infusion [54]. Muco et al. (2022) reported severe outcomes resulting from delayed management of vasospastic angina symptoms. The patient’s cardiac evaluation was performed 3 days after the onset of symptoms, and unfortunately, she did not recover from brain damage caused by coronary vasospastic sequelae. The authors stressed the importance of medical teams recognizing the symptoms of CRCT through vigilant monitoring and patient education [55].

As seen in the symptoms of CRCT caused by heart failure and vasospasm, careful observation of symptoms and conducting appropriate tests are crucial to prevent cardiotoxicity and minimize damage. These characteristics of CRCT and the associated symptoms related to chemotherapy regimens can provide crucial educational content for healthcare providers and patients preparing for chemotherapy. In addition, CRCT and symptom progression according to chemotherapy regimens could be used to formulate research questions for future systematic reviews.

Second, the preventive management of CRCT necessitates adherence to recommended guidelines. The 2022 ESC guidelines on cardio-oncology have updated the classification of CRCT and the monitoring protocols based on the chemotherapy regimens used [7]. The CRCT identified in the current study aligns with the drug toxicity outlined in the 2022 ESC guidelines. These guidelines advocate for regular cardiac monitoring before, during, and after chemotherapy to prevent and manage CRCT induced by anthracycline and HER2 blockers [7, 12]. In this scoping review, two of 23 records described cardiac monitoring before, during, and after chemotherapy. An Australian multicenter study revealed that 59% of patients were referred to a cardiologist before CRCT occurred, but only 15% of patients diagnosed with CRCT had consulted a cardiologist before chemotherapy [41]. Given the declining mortality rates among cancer patients, managing CRCT requires a collaborative approach between oncology and cardiology to minimize mortality and morbidity in patients with breast cancer undergoing chemotherapy [7]. Therefore, it remains crucial to emphasize adherence to cardiac monitoring guidelines and foster cooperation between oncology and cardiology.

Additionally, symptom assessment is important for the early detection of patients with CRCT. The studies included in the current scoping review assessed whether patients’ symptoms could detect CRCT using interviews with patients, the New York Heart Association classification, a dyspnea assessment scale, and CTCAE tools. The United States National Cancer Institute recommends that healthcare providers use CTCAE and patients with cancer use PRO to report adverse events, including symptoms. CTCAE is a broad and comprehensive terminology that encompasses adverse events related to cancer treatment, has been used since the 1980s [25], and is not specialized in cardiotoxicity. Additionally, a discrepancy between CTCAE and PRO discovered that healthcare providers often underestimate both the incidence and duration of symptoms compared to the patients [56–58]. Specifically, healthcare providers tend to report symptom severity as lower than that reported by patients. For instance, there are notable discrepancies between healthcare providers and patients when reporting severe or very severe symptoms of fatigue, dyspnea, and limb edema in patients with early-stage breast cancer undergoing chemotherapy. The reported rates were 8% and 22% for fatigue, 0% and 4% for dyspnea, and 0% and 5% for limb edema, from healthcare providers and patients, respectively. Therefore, it is necessary to develop a user-friendly questionnaire to assess the various symptoms of CRCT.

Finally, we found that once CRCT was confirmed, cardiac treatment was promptly initiated and chemotherapy was frequently halted until CRCT resolution. A Delphi study on the use of anthracycline and trastuzumab proposed altering the treatment schedule or discontinuing treatment until there was an improvement in LVEF [59]. However, the professional societies did not provide definitive recommendations regarding continuing or ceasing ongoing chemotherapy. Instead, they suggested that the decision to continue or discontinue ongoing chemotherapy should be made based on the patient’s potential risks and benefits [60]. For example, Polk et al. (2016) reported that out of 22 patients with CRCT resulting from capecitabine, six continued medications with or without cardiac treatment; some of these patients experienced the same symptoms, while others did not exhibit significant symptoms [40]. Further research is required to explore the continuation or discontinuation of chemotherapy when CRCT is confirmed.

This study has some limitations. First, although we did not restrict the patients’ sex when reviewing the literature, most patients, except for one, were female. This may be related to the lower incidence of breast cancer in men. Second, although this scoping review mapped CRCT symptoms according to chemotherapy regimens, including anthracycline-based drugs, HER2 blockers, and antimetabolites, it did not cover cardiotoxicity related to other types of chemotherapy regimens. Thus, exploring the symptoms by focusing on expanded chemotherapy regimens and cardiovascular toxic diseases will assist in overcoming this limitation. Third, of the 29 studies, 23 were case reports with some grey literature, which may be justified by the nature of scoping reviews that allow for inclusion irrespective of the data source [61] and the study type. Experimental or observational clinical studies use objective criteria, such as diagnostic tests to generate primary evidence. However, case reports have led to new medical discoveries regarding the prevention and treatment of diseases [62]. Given the nature of case reports, specific symptoms that could provide clues for evaluating CRCT in patients with breast cancer are most often found in these reports. We incorporated grey literature to gather more comprehensive information on CRCT-related symptoms. However, to mitigate the potential issue of unverified quality in grey literature, we initially organized 16 studies from peer-reviewed literature and subsequently incorporated the grey literature into our findings. This approach helped to clarify the results of the peer-reviewed literature, particularly the types of chemotherapy regimens [63]. Finally, regarding the literature selection criteria, we examined articles written in English and published since 2010, the year the cardio-oncology guidelines were announced, thereby excluding articles published before 2010.

## Conclusion

This scoping review allowed data mapping according to the study design and chemotherapy regimens. The key messages included a type of CRCT, cardiac assessment, and in-depth information regarding the CRCT symptoms. There were approximately five typical CRCT symptoms, including dyspnea, chest pain, peripheral edema, fatigue, and palpitations, and the timing of symptom occurrence varied. The symptoms were assessed by patient-reported symptom presentation rather than using a symptom assessment tool. Therefore, developing and applying a CRCT-specific and user-friendly symptom assessment tool are expected to help healthcare providers and patients manage CRCT symptoms effectively.

### Supplementary Information

Supplementary Material 1.

Supplementary Material 2.

## Data Availability

The datasets generated during and/or analyzed during the current study are available from the corresponding author upon reasonable request.

## References

[1] Arnold M, Morgan E, Rumgay H, Mafra A, Singh D, Laversanne M, Vignat J, Gralow JR, Cardoso F, Siesling S, Soerjomataram I (2022). Current and future burden of breast cancer: global statistics for 2020 and 2040. The Breast.

[2] Siegel RL, Miller KD, Wagle NS, Jemal A (2023). Cancer statistics, 2023. CA Cancer J Clin.

[3] Agha A, Wang X, Wang M, Lehrer EJ, Horn SR, Rosenberg JC, Trifiletti DM, Diaz R, Louie AV, Zaorsky NG (2022). Long-term risk of death from heart disease among breast cancer patients. Front Cardiovasc Med.

[4] Oikawa M, Ishida T, Takeishi Y (2023). Cancer therapeutics-related cardiovascular dysfunction: Basic mechanisms and clinical manifestation. J Cardiol.

[5] Piepoli MF, Adamo M, Barison A, Bestetti RB, Biegus J, Böhm M, Butler J, Carapetis J, Ceconi C, Chioncel O (2022). Preventing heart failure: a position paper of the Heart Failure Association in collaboration with the European Association of Preventive Cardiology. Eur J Heart Fail.

[6] Chung R, Ghosh AK, Banerjee A: Cardiotoxicity: precision medicine with imprecise definitions. In., vol. 5: Archives of Disease in childhood; 2018: e000774. 10.1136/openhrt-2018-000774 PMC6074618 30094034

[7] Lyon AR, López-Fernández T, Couch LS, Asteggiano R, Aznar MC, Bergler-Klein J, Boriani G, Cardinale D, Cordoba R, Cosyns B (2022). 2022 ESC Guidelines on cardio-oncology developed in collaboration with the European Hematology Association (EHA), the European Society for Therapeutic Radiology and Oncology (ESTRO) and the International Cardio-Oncology Society (IC-OS): developed by the task force on cardio-oncology of the European Society of Cardiology (ESC). European Heart Journal - Cardiovascular Imaging.

[8] McDonagh TA, Metra M, Adamo M, Gardner RS, Baumbach A, Böhm M, Burri H, Butler J, Čelutkienė J, Chioncel O et al: 2021 ESC Guidelines for the diagnosis and treatment of acute and chronic heart failure: developed by the Task Force for the diagnosis and treatment of acute and chronic heart failure of the European Society of Cardiology (ESC). With the special contribution of the Heart Failure Association (HFA) of the ESC. Eur J Heart Fail 2022, 24(1):4-131. 10.1002/ejhf.2333 35083827

[9] Armenian SH, Lacchetti C, Lenihan D (2017). Prevention and monitoring of cardiac dysfunction in survivors of adult cancers: American Society of Clinical Oncology Clinical Practice Guideline Summary. J Oncol Pract.

[10] Lanza O, Ferrera A, Reale S, Solfanelli G, Petrungaro M, Tini Melato G, Volpe M, Battistoni A: New insights on the toxicity on heart and vessels of breast cancer therapies. Med Sci (Basel) 2022, 10(2). 10.3390/medsci10020027 PMC9229896 35736347

[11] Runowicz CD, Leach CR, Henry NL, Henry KS, Mackey HT, Cowens-Alvarado RL, Cannady RS, Pratt-Chapman ML, Edge SB, Jacobs LA (2016). American Cancer Society/American Society of Clinical Oncology Breast Cancer Survivorship Care Guideline. CA Cancer J Clin.

[12] Lee GA, Aktaa S, Baker E, Gale CP, Yaseen IF, Gulati G, Asteggiano R, Szmit S, Cohen-Solal A, Abdin A (2022). European Society of Cardiology quality indicators for the prevention and management of cancer therapy-related cardiovascular toxicity in cancer treatment. Eur Heart J Qual Care Clin Outcomes.

[13] Alexandraki A, Papageorgiou E, Zacharia M, Keramida K, Papakonstantinou A, Cipolla CM, Tsekoura D, Naka K, Mazzocco K, Mauri D et al: New insights in the era of clinical biomarkers as potential predictors of systemic therapy-induced cardiotoxicity in women with breast cancer: a systematic review. Cancers (Basel) 2023, 15(13). 10.3390/cancers15133290 PMC10340234 37444400

[14] Di Lisi D, Manno G, Madaudo C, Filorizzo C, Intravaia RCM, Galassi AR, Incorvaia L, Russo A, Novo G: Chemotherapy-related cardiac dysfunction: the usefulness of myocardial work indices. Int J Cardiovasc Imaging 2023. 10.1007/s10554-023-02897-9 37548845

[15] Kar J, Cohen MV, McQuiston SA, Malozzi CM (2023). Can global longitudinal strain (GLS) with magnetic resonance prognosticate early cancer therapy-related cardiac dysfunction (CTRCD) in breast cancer patients, a prospective study?. Magn Reson Imaging.

[16] Lim A, Jang H, Jeon M, Fadol AP, Kim S (2022). Cancer treatment-related cardiac dysfunction in breast cancer survivors: a retrospective descriptive study using electronic health records from a Korean tertiary hospital. Eur J Oncol Nurs.

[17] Liu W, Li W, Li H, Li Z, Zhao P, Guo Z, Liu C, Sun L, Wang Z (2022). Two-dimensional speckle tracking echocardiography help identify breast cancer therapeutics-related cardiac dysfunction. BMC Cardiovasc Disord.

[18] Mauro C, Capone V, Cocchia R, Cademartiri F, Riccardi F, Arcopinto M, Alshahid M, Anwar K, Carafa M, Carbone A et al: Cardiovascular side effects of anthracyclines and HER2 inhibitors among patients with breast cancer: a multidisciplinary stepwise approach for prevention, early detection, and treatment. J Clin Med 2023, 12(6). 10.3390/jcm12062121 PMC10056500 36983126

[19] Okushi Y, Saijo Y, Yamada H, Toba H, Zheng R, Seno H, Takahashi T, Ise T, Yamaguchi K, Yagi S et al: Effectiveness of surveillance by echocardiography for cancer therapeutics-related cardiac dysfunction of patients with breast cancer. J Cardiol 2023. 10.1016/j.jjcc.2023.07.002 37481235

[20] Ositelu K, Trevino A, Tong A, Chen MH, Akhter N: Challenges in cardiovascular imaging in women with breast cancer. Curr Cardiol Rep 2023. 10.1007/s11886-023-01941-3 37642930

[21] Terui Y, Sugimura K, Ota H, Tada H, Nochioka K, Sato H, Katsuta Y, Fujiwara J, Harada-Shoji N, Sato-Tadano A (2023). Usefulness of cardiac magnetic resonance for early detection of cancer therapeutics-related cardiac dysfunction in breast cancer patients. Int J Cardiol.

[22] Thavendiranathan P, Shalmon T, Fan CS, Houbois C, Amir E, Thevakumaran Y, Somerset E, Malowany JM, Urzua-Fresno C, Yip P (2023). Comprehensive cardiovascular magnetic resonance tissue characterization and cardiotoxicity in women with breast cancer. JAMA Cardiol.

[23] Trotti A, Colevas AD, Setser A, Basch E (2007). Patient-reported outcomes and the evolution of adverse event reporting in oncology. J Clin Oncol.

[24] White J, Byles J, Williams T, Untaru R, Ngo DTM, Sverdlov AL (2022). Early access to a cardio-oncology clinic in an Australian context: a qualitative exploration of patient experiences. Cardiooncology.

[25] Trotti A, Colevas AD, Setser A, Rusch V, Jaques D, Budach V, Langer C, Murphy B, Cumberlin R, Coleman CN, Rubin P: CTCAE v3.0: development of a comprehensive grading system for the adverse effects of cancer treatment. Semin Radiat Oncol 2003, 13(3):176-181. 10.1016/S1053-4296(03)00031-6 12903007

[26] Basch E, Reeve BB, Mitchell SA, Clauser SB, Minasian LM, Dueck AC, Mendoza TR, Hay J, Atkinson TM, Abernethy AP et al: Development of the National Cancer Institute’s patient-reported outcomes version of the common terminology criteria for adverse events (PRO-CTCAE). J Natl Cancer Inst 2014, 106(9). 10.1093/jnci/dju244 PMC4200059 25265940

[27] Kluetz PG, Chingos DT, Basch EM, Mitchell SA (2016). Patient-reported outcomes in cancer clinical trials: measuring symptomatic adverse events with the National Cancer Institute’s Patient-Reported Outcomes Version of the Common Terminology Criteria for Adverse Events (PRO-CTCAE). Am Soc Clin Oncol Educ Book.

[28] Basch E, Deal AM, Dueck AC, Scher HI, Kris MG, Hudis C, Schrag D (2017). Overall survival results of a trial assessing patient-reported outcomes for symptom monitoring during routine cancer treatment. JAMA.

[29] Liu L, Suo T, Shen Y, Geng C, Song Z, Liu F, Wang J, Xie Y, Zhang Y, Tang T (2020). Clinicians versus patients subjective adverse events assessment: based on patient-reported outcomes version of the common terminology criteria for adverse events (PRO-CTCAE). Qual Life Res.

[30] Munn Z, Pollock D, Khalil H, Alexander L, Mclnerney P, Godfrey CM, Peters M, Tricco AC (2022). What are scoping reviews? Providing a formal definition of scoping reviews as a type of evidence synthesis. JBI Evidence Synthesis.

[31] Arksey H, O’Malley L (2005). Scoping studies: towards a methodological framework. International Journal of Social Research Methodology.

[32] Peters M, Godfrey C, McInerney P, Munn Z, Tricco A, Khalil H: Chapter 11: scoping reviews (2020 version). In: JBI Manual for Evidence Synthesis. edn. Edited by Aromataris E MZ: JBI; 2020.

[33] Munn Z, Peters MD, Stern C, Tufanaru C, McArthur A, Aromataris E (2018). Systematic review or scoping review? Guidance for authors when choosing between a systematic or scoping review approach. BMC medical research methodology.

[34] Peters MDJ, Godfrey C, McInerney P, Khalil H, Larsen P, Marnie C, Pollock D, Tricco AC, Munn Z (2022). Best practice guidance and reporting items for the development of scoping review protocols. JBI Evid Synth.

[35] Bohdan M, Kowalczys A, Mickiewicz A, Gruchala M, Lewicka E: Cancer therapy-related cardiovascular complications in clinical practice: current perspectives. J Clin Med 2021, 10(8). 10.3390/jcm10081647 PMC8069381 33924543

[36] Priori SG, Wilde AA, Horie M, Cho Y, Behr ER, Berul C, Blom N, Brugada J, Chiang CE, Huikuri H (2013). HRS/EHRA/APHRS expert consensus statement on the diagnosis and management of patients with inherited primary arrhythmia syndromes: document endorsed by HRS, EHRA, and APHRS in May 2013 and by ACCF, AHA, PACES, and AEPC in June 2013. Heart Rhythm.

[37] Bozkurt B, Coats A, Tsutsui H: Universal definition and classification of heart failure. J Card Fail 2021.

[38] Aldiab A (2010). Cardiotoxicity with adjuvant trastuzumab use in breast cancer: a single institution»s experience. J Saudi Heart Assoc.

[39] Russell SD, Blackwell KL, Lawrence J, Pippen JE, Roe MT, Wood F, Paton V, Holmgren E, Mahaffey KW (2010). Independent adjudication of symptomatic heart failure with the use of doxorubicin and cyclophosphamide followed by trastuzumab adjuvant therapy: a combined review of cardiac data from the National Surgical Adjuvant Breast and Bowel Project B-31 and the North Central Cancer Treatment Group N9831 clinical trials. J Clin Oncol.

[40] Polk A, Shahmarvand N, Vistisen K, Vaage-Nilsen M, Larsen FO, Schou M, Nielsen DL: Incidence and risk factors for capecitabine-induced symptomatic cardiotoxicity: a retrospective study of 452 consecutive patients with metastatic breast cancer. BMJ Open 2016, 6(10). 10.1136/bmjopen-2016-012798 PMC5073470 27798021

[41] Clark RA, Marin TS, McCarthy AL, Bradley J, Grover S, Peters R, Karapetis CS, Atherton JJ, Koczwara B (2019). Cardiotoxicity after cancer treatment: a process map of the patient treatment journey. Cardiooncology.

[42] Anjos M, Fontes-Oliveira M, Costa VM, Santos M, Ferreira R (2021). An update of the molecular mechanisms underlying doxorubicin plus trastuzumab induced cardiotoxicity. Life Sci.

[43] Malik A, Brito D, Vaqar S, Chhabra L: Congestive heart failure. In: StatPearls. edn. Treasure Island (FL): StatPearls Publishing. Copyright © 2023, StatPearls Publishing LLC.; 2023.

[44] Attin M, Reifenstein K, Mehta S, Arcoleo K, Lin CD, Storozynsky E (2022). Reported signs, symptoms, and diagnostic tests before cardiotoxicity among women with breast cancer: a pilot study. J Cardiovasc Nurs.

[45] Huang P, Dai S, Ye Z, Liu Y, Chen Z, Zheng Y, Shao X, Lei L, Wang X (2017). Long-term tolerance and cardiac function in breast cancer patients receiving trastuzumab therapy. Oncotarget.

[46] Salyer J, Flattery M, Lyon DE (2019). Heart failure symptom clusters and quality of life. Heart Lung.

[47] Padegimas A, Carver JR (2020). How to diagnose and manage patients with fluoropyrimidine-induced chest pain: a single center approach. JACC CardioOncol.

[48] Sheth MA, Widmer RJ, Dandapantula HK (2021). Pathobiology and evolving therapies of coronary artery vasospasm. Proc (Bayl Univ Med Cent).

[49] Hokimoto S, Kaikita K, Yasuda S, Tsujita K, Ishihara M, Matoba T, Matsuzawa Y, Mitsutake Y, Mitani Y, Murohara T (2023). JCS/CVIT/JCC 2023 guideline focused update on diagnosis and treatment of vasospastic angina (coronary spastic angina) and coronary microvascular dysfunction. J Cardiol.

[50] Kanduri J, More LA, Godishala A, Asnani A (2019). Fluoropyrimidine-associated cardiotoxicity. Cardiol Clin.

[51] Garbis K, Rafiee MJ, Luu J (2023). 5-fluorouracil-induced coronary vasospasm: a cardiovascular magnetic resonance imaging case report. Glob Cardiol Sci Pract.

[52] Dyhl-Polk A, Vaage-Nilsen M, Schou M, Vistisen KK, Lund CM, Kümler T, Appel JM, Nielsen DL (2020). Incidence and risk markers of 5-fluorouracil and capecitabine cardiotoxicity in patients with colorectal cancer. Acta Oncol.

[53] Becker K, Erckenbrecht JF, Häussinger D, Fueling T (1999). Cardiotoxicity of the antiprolif erative compound fluorouracil. Drugs.

[54] Lestuzzi C, Vaccher E, Talamini R, Lleshi A, Meneguzzo N, Viel E, Scalone S, Tartuferi L, Buonadonna A, Ejiofor L, Schmoll HJ (2014). Effort myocardial ischemia during chemotherapy with 5-fluorouracil: an underestimated risk. Ann Oncol.

[55] Muco E, Patail H, Shaik A, McMahon S (2022). Capecitabine-associated coronary vasospasm and cardiac arrest. Cureus.

[56] Montemurro F, Mittica G, Cagnazzo C, Longo V, Berchialla P, Solinas G, Culotta P, Martinello R, Foresto M, Gallizioli S (2016). Self-evaluation of adjuvant chemotherapy-related adverse effects by patients with breast cancer. JAMA Oncol.

[57] Atkinson TM, Ryan SJ, Bennett AV, Stover AM, Saracino RM, Rogak LJ, Jewell ST, Matsoukas K, Li Y, Basch E (2016). The association between clinician-based common terminology criteria for adverse events (CTCAE) and patient-reported outcomes (PRO): a systematic review. Support Care Cancer.

[58] Galizia D, Milani A, Geuna E, Martinello R, Cagnazzo C, Foresto M, Longo V, Berchialla P, Solinas G, Calori A (2018). Self-evaluation of duration of adjuvant chemotherapy side effects in breast cancer patients: a prospective study. Cancer Med.

[59] Gavila J, Seguí M, Calvo L, López T, Alonso JJ, Farto M (2017). Sánchez-de la Rosa R: Evaluation and management of chemotherapy-induced cardiotoxicity in breast cancer: a Delphi study. Clin Transl Oncol.

[60] Leong DP, Lenihan DJ (2022). Clinical practice guidelines in cardio-oncology. Heart Fail Clin.

[61] Munn Z, Pollock D, Khalil H, Alexander L, McLnerney P, Godfrey CM, Peters M, Tricco AC (2022). What are scoping reviews? Providing a formal definition of scoping reviews as a type of evidence synthesis. JBI Evid Synth.

[62] Li YR, Jia Z, Zhu H (2013). Understanding the value of case reports and studies in the context of clinical research, research design and evidence-based practice. J Case Reports and Studies.

[63] Conn VS, Valentine JC, Cooper HM, Rantz MJ (2003). Grey literature in meta-analyses. Nurs Res.

