# Supplementary material for: Chemotherapy-related cardiotoxicity and its symptoms in patients with breast cancer: a scoping review
Source: Syst Rev. 2024 Jun 27;13:167. doi: 10.1186/s13643-024-02588-z (PMC11212164; doi:10.1186/s13643-024-02588-z)
Supplement: Supplementary file 1 — Supplementary Material 1. [file 13643_2024_2588_MOESM1_ESM.docx]

**Scoping Review protocol**

**1. Title**

Chemotherapy-related cardiotoxicity and its symptoms in patients with breast cancer: A scoping review

**2. Objective**

This scoping review was conducted to explore the scope of research focused on CRCT symptoms, including the general characteristics of the studies, diagnostic tools and monitoring practices related to detecting CRCT, and the characteristics and progression of symptoms associated with CRCT.

**3. Review questions**

1) What are the general characteristics of the studies related to CRCT in patients with breast cancer?

2) What diagnostic tools and monitoring practices are used to detect CRCT?

3) What are the characteristics and progression of symptoms associated with CRCT?

**4. Introduction**

Breast cancer is currently the most common cancer worldwide. Its incidence and mortality rates in East Asia in 2020 accounted for 24% and 20% of the global rates, respectively, and these rates are expected to continue increasing until 2040 [1]. In the United States, since the mid-2000s, the incidence rate of breast cancer has been increasing by 0.5% annually, while the mortality rate has been decreasing by 1% per year from 2011 to 2020 [2]. Despite the improved long-term survival rate in patients with breast cancer due to the development of chemotherapy, the literature has highlighted that cardiotoxicity, a cardiac problem caused by chemotherapy, could be a significant cause of death among these patients [3]. Chemotherapy-Related CardioToxicity (CRCT) can interfere with cancer treatment and progress to congestive heart failure during or after chemotherapy [4], potentially lowering the survival rate and quality of life of patients with cancer [5].

The term cardiotoxicity was first used in the 1970s to describe cardiac complications resulting from chemotherapy regimens, such as anthracyclines and 5-fluorouracil. The early definition of cardiotoxicity centered around heart failure, but the current definition is broad and still imprecise [6]. The 2022 guidelines on cardio-oncology from the European Society of Cardiology (ESC) defines cardiotoxicity as including cardiac dysfunction, myocarditis, vascular toxicity, arterial hypertension, and cardiac arrhythmias. Some of these definitions reflect the symptoms. For example, cardiac dysfunction, which accounts for 48% of cardiotoxicity in patients with cancer, is divided into asymptomatic and symptomatic cardiac dysfunction. Asymptomatic cardiac dysfunction is defined based on left ventricular ejection fraction (LVEF), myocardial global longitudinal strain, and cardiac biomarkers. Symptomatic cardiac dysfunction indicates heart failure and presents with ankle swelling, breathlessness, and fatigue [7]. The ESC guidelines for heart failure present more than 20 types of symptoms [8]; however, to the best of our knowledge, few studies have been conducted to determine which heart failure symptoms and their characteristics are associated with CRCT in patients with breast cancer. Similarly, there is a lack of information related to vascular toxicity such as myocardial infarction [7].

Professional societies in cardiology and oncology have proposed guidelines for the prevention and management of cardiotoxicity in patients with cancer. According to the American Society of Clinical Oncology and the ESC, it is recommended to identify high-risk patients, comprehensively evaluate clinical signs and symptoms associated with CRCT, and conduct cardiac evaluations before, during, and after chemotherapy [7, 9, 10]. In addition, guidelines for patients with cancer, including those for breast cancer survivorship care, emphasize that patients should be aware of the potential risk of CRCT and report symptoms, such as fatigue or shortness of breath to their healthcare providers [7, 11, 12]. Although these guidelines encompass cardiac monitoring as well as symptom observation, many studies have focused solely on objective diagnostic tests, such as echocardiography, cardiac magnetic resonance, and cardiac biomarkers [13-22], which means that there is little interest in CRCT symptoms in patients under breast care.

This lack of interest in CRCT symptoms may be related to the absence of a specific symptom assessment tool for CRCT. Symptom monitoring of CRCT in patients with breast cancer was conducted through patient interviews and reported using the appropriate terminology [23]. In terms of interviews, patients with cancer experienced the burden of expressing symptoms between cardiovascular problems and cancer treatment. Qualitative research on patients with cancer indicates that these patients experience a daily battle to distinguish the symptoms they experience during chemotherapy [24]. To reduce the burden of identifying CRCT symptoms, it is crucial to educate patients with breast cancer undergoing chemotherapy about these symptoms. To report cardiotoxicity, healthcare providers in oncology can use a dictionary of terms called the Common Terminology Criteria for Adverse Events (CTCAE) for reporting adverse events in patients with cancer [25]. Patients can also use Patient-Reported Outcome (PRO), which allows unfiltered reporting of symptoms directly to the clinical database [26]. PRO consists of 78 symptomatic adverse events out of approximately 1,000 types of CTCAE [27]. Basch et al. suggested that PRO could enable healthcare providers to identify patient symptoms before they worsen, thereby improving the overall survival rate of patients with metastatic cancer [28]. This finding implies that symptoms can provide valuable clues for enhancing the timeliness and accuracy of clinical assessments of CRCT [29]. Therefore, it is necessary to explore the scope of research focusing on CRCT symptoms for prevention and early detection of CRCT in patients with breast cancer.

**5. Eligibility criteria**

**Participants**

All the breast cancer patients

**Concept**

1) Studies that specifically reported symptoms directly matched to CRCT in breast cancer patients.

2) Literature published in English since 2010, in line with the year the CRCT guidelines were announced by the Cardio-Oncology Society.

**Context**

Open

**Types of evidence sources**

All kinds of research designs

**Exclusion criteria**

1) Studies that included patients with other types of cancer

2) Studies that involved animal subjects

3) Studies that reported symptoms not directly related to CRCT

**6. Method**

**Search Queries (Supplementary Table 1)**

**Database**

1) PubMed, CINAHL, and Embase

**7****. Source of evidence selection**

1) Review of titles and abstracts according to the inclusion criteria

2) Full text review of the selected studies according to the inclusion criteria

**8. Data extraction**

1) General characteristics of study: author, publication year, country of origin, study design

2) Population information: sample size, sex, age, cancer type, cancer stage

3) Chemotherapy information: chemotherapy regimen

4) Cardiotoxicity information: type of cardiotoxicity, diagnostic tests, times of assessment

5) Symptom information: type of symptom, characteristic (including symptom worsening or improvement), onset time, progression time, time to symptom improved

6) Whether to receive chemotherapy after diagnosis of cardiotoxicity

7) Any other key findings related to symptoms associated with CRCT

**8.** **Data analysis and presentation**

The content from the included studies is divided into three mappings: 1) general characteristics, which encompassed study designs, patients, and medications; 2) diagnostic tools and monitoring practices for CRCT; and 3) the characteristics and progression of symptoms associated with CRCT. We expect to find information about CRCT symptoms-related data and present them with Tables and Figures.

**9. Deviation from the protocol**

If a deviation occurs, it will be mentioned in the manuscript to ensure transparency.

**References**

1. Arnold M, Morgan E, Rumgay H, Mafra A, Singh D, Laversanne M, Vignat J, Gralow JR, Cardoso F, Siesling S, Soerjomataram I: **Current and future burden of breast cancer: Global statistics for 2020 and 2040**. *The Breast* 2022, **66**:15-23.

2. Siegel RL, Miller KD, Wagle NS, Jemal A: **Cancer statistics, 2023**. *CA Cancer J Clin* 2023, **73**(1):17-48.

3. Agha A, Wang X, Wang M, Lehrer EJ, Horn SR, Rosenberg JC, Trifiletti DM, Diaz R, Louie AV, Zaorsky NG: **Long-Term Risk of Death From Heart Disease Among Breast Cancer Patients**. *Front Cardiovasc Med* 2022, **9**:784409.

4. Oikawa M, Ishida T, Takeishi Y: **Cancer therapeutics-related cardiovascular dysfunction: Basic mechanisms and clinical manifestation**. *J Cardiol* 2023, **81**(3):253-259.

5. Piepoli MF, Adamo M, Barison A, Bestetti RB, Biegus J, Böhm M, Butler J, Carapetis J, Ceconi C, Chioncel O *et al*: **Preventing heart failure: a position paper of the Heart Failure Association in collaboration with the European Association of Preventive Cardiology**. *Eur J Heart Fail* 2022, **24**(1):143-168.

6. Chung R, Ghosh AK, Banerjee A: **Cardiotoxicity: precision medicine with imprecise definitions**. In*.*, vol. 5: Archives of Disease in childhood; 2018: e000774.

7. Lyon AR, López-Fernández T, Couch LS, Asteggiano R, Aznar MC, Bergler-Klein J, Boriani G, Cardinale D, Cordoba R, Cosyns B *et al*: **2022 ESC Guidelines on cardio-oncology developed in collaboration with the European Hematology Association (EHA), the European Society for Therapeutic Radiology and Oncology (ESTRO) and the International Cardio-Oncology Society (IC-OS): Developed by the task force on cardio-oncology of the European Society of Cardiology (ESC)**. *European Heart Journal - Cardiovascular Imaging* 2022, **23**(10):e333-e465.

8. McDonagh TA, Metra M, Adamo M, Gardner RS, Baumbach A, Böhm M, Burri H, Butler J, Čelutkienė J, Chioncel O *et al*: **2021 ESC Guidelines for the diagnosis and treatment of acute and chronic heart failure: Developed by the Task Force for the diagnosis and treatment of acute and chronic heart failure of the European Society of Cardiology (ESC). With the special contribution of the Heart Failure Association (HFA) of the ESC**. *Eur J Heart Fail* 2022, **24**(1):4-131.

9. Armenian SH, Lacchetti C, Lenihan D: **Prevention and Monitoring of Cardiac Dysfunction in Survivors of Adult Cancers: American Society of Clinical Oncology Clinical Practice Guideline Summary**. *J Oncol Pract* 2017, **13**(4):270-275.

10. Lanza O, Ferrera A, Reale S, Solfanelli G, Petrungaro M, Tini Melato G, Volpe M, Battistoni A: **New Insights on the Toxicity on Heart and Vessels of Breast Cancer Therapies**. *Med Sci (Basel)* 2022, **10**(2).

11. Runowicz CD, Leach CR, Henry NL, Henry KS, Mackey HT, Cowens-Alvarado RL, Cannady RS, Pratt-Chapman ML, Edge SB, Jacobs LA *et al*: **American Cancer Society/American Society of Clinical Oncology Breast Cancer Survivorship Care Guideline**. *CA Cancer J Clin* 2016, **66**(1):43-73.

12. Lee GA, Aktaa S, Baker E, Gale CP, Yaseen IF, Gulati G, Asteggiano R, Szmit S, Cohen-Solal A, Abdin A *et al*: **European Society of Cardiology quality indicators for the prevention and management of cancer therapy-related cardiovascular toxicity in cancer treatment**. *Eur Heart J Qual Care Clin Outcomes* 2022, **9**(1):1-7.

13. Alexandraki A, Papageorgiou E, Zacharia M, Keramida K, Papakonstantinou A, Cipolla CM, Tsekoura D, Naka K, Mazzocco K, Mauri D *et al*: **New Insights in the Era of Clinical Biomarkers as Potential Predictors of Systemic Therapy-Induced Cardiotoxicity in Women with Breast Cancer: A Systematic Review**. *Cancers (Basel)* 2023, **15**(13).

14. Di Lisi D, Manno G, Madaudo C, Filorizzo C, Intravaia RCM, Galassi AR, Incorvaia L, Russo A, Novo G: **Chemotherapy-related cardiac dysfunction: the usefulness of myocardial work indices**. *Int J Cardiovasc Imaging* 2023.

15. Kar J, Cohen MV, McQuiston SA, Malozzi CM: **Can global longitudinal strain (GLS) with magnetic resonance prognosticate early cancer therapy-related cardiac dysfunction (CTRCD) in breast cancer patients, a prospective study?** *Magn Reson Imaging* 2023, **97**:68-81.

16. Lim A, Jang H, Jeon M, Fadol AP, Kim S: **Cancer treatment-related cardiac dysfunction in breast cancer survivors: A retrospective descriptive study using electronic health records from a Korean tertiary hospital**. *Eur J Oncol Nurs* 2022, **59**:102163.

17. Liu W, Li W, Li H, Li Z, Zhao P, Guo Z, Liu C, Sun L, Wang Z: **Two-dimensional speckle tracking echocardiography help identify breast cancer therapeutics-related cardiac dysfunction**. *BMC Cardiovasc Disord* 2022, **22**(1):548.

18. Mauro C, Capone V, Cocchia R, Cademartiri F, Riccardi F, Arcopinto M, Alshahid M, Anwar K, Carafa M, Carbone A *et al*: **Cardiovascular Side Effects of Anthracyclines and HER2 Inhibitors among Patients with Breast Cancer: A Multidisciplinary Stepwise Approach for Prevention, Early Detection, and Treatment**. *J Clin Med* 2023, **12**(6).

19. Okushi Y, Saijo Y, Yamada H, Toba H, Zheng R, Seno H, Takahashi T, Ise T, Yamaguchi K, Yagi S *et al*: **Effectiveness of surveillance by echocardiography for cancer therapeutics-related cardiac dysfunction of patients with breast cancer**. *J Cardiol* 2023.

20. Ositelu K, Trevino A, Tong A, Chen MH, Akhter N: **Challenges in Cardiovascular Imaging in Women with Breast Cancer**. *Curr Cardiol Rep* 2023.

21. Terui Y, Sugimura K, Ota H, Tada H, Nochioka K, Sato H, Katsuta Y, Fujiwara J, Harada-Shoji N, Sato-Tadano A *et al*: **Usefulness of cardiac magnetic resonance for early detection of cancer therapeutics-related cardiac dysfunction in breast cancer patients**. *Int J Cardiol* 2023, **371**:472-479.

22. Thavendiranathan P, Shalmon T, Fan CS, Houbois C, Amir E, Thevakumaran Y, Somerset E, Malowany JM, Urzua-Fresno C, Yip P *et al*: **Comprehensive Cardiovascular Magnetic Resonance Tissue Characterization and Cardiotoxicity in Women With Breast Cancer**. *JAMA Cardiol* 2023, **8**(6):524-534.

23. Trotti A, Colevas AD, Setser A, Basch E: **Patient-reported outcomes and the evolution of adverse event reporting in oncology**. *J Clin Oncol* 2007, **25**(32):5121-5127.

24. White J, Byles J, Williams T, Untaru R, Ngo DTM, Sverdlov AL: **Early access to a cardio-oncology clinic in an Australian context: a qualitative exploration of patient experiences**. *Cardiooncology* 2022, **8**(1):14.

25. Trotti A, Colevas AD, Setser A, Rusch V, Jaques D, Budach V, Langer C, Murphy B, Cumberlin R, Coleman CN, Rubin P: **CTCAE v3.0: development of a comprehensive grading system for the adverse effects of cancer treatment**. *Semin Radiat Oncol* 2003, **13**(3):176-181.

26. Basch E, Reeve BB, Mitchell SA, Clauser SB, Minasian LM, Dueck AC, Mendoza TR, Hay J, Atkinson TM, Abernethy AP *et al*: **Development of the National Cancer Institute's patient-reported outcomes version of the common terminology criteria for adverse events (PRO-CTCAE)**. *J Natl Cancer Inst* 2014, **106**(9).

27. Kluetz PG, Chingos DT, Basch EM, Mitchell SA: **Patient-reported outcomes in cancer clinical trials: measuring symptomatic adverse events with the National Cancer Institute’s Patient-Reported Outcomes Version of the Common Terminology Criteria for Adverse Events (PRO-CTCAE)**. *Am Soc Clin Oncol Educ Book* 2016, **36**:67-73.

28. Basch E, Deal AM, Dueck AC, Scher HI, Kris MG, Hudis C, Schrag D: **Overall Survival Results of a Trial Assessing Patient-Reported Outcomes for Symptom Monitoring During Routine Cancer Treatment**. *JAMA* 2017, **318**(2):197-198.

29. Liu L, Suo T, Shen Y, Geng C, Song Z, Liu F, Wang J, Xie Y, Zhang Y, Tang T *et al*: **Clinicians versus patients subjective adverse events assessment: based on patient-reported outcomes version of the common terminology criteria for adverse events (PRO-CTCAE)**. *Qual Life Res* 2020, **29**(11):3009-3015.
