# Supplementary material for: Chemotherapy-related cardiotoxicity and its symptoms in patients with breast cancer: a scoping review
Source: Syst Rev. 2024 Jun 27;13:167. doi: 10.1186/s13643-024-02588-z (PMC11212164; doi:10.1186/s13643-024-02588-z)
Supplement: Supplementary file 2 — Supplementary Material 2. [file 13643_2024_2588_MOESM2_ESM.docx]

**Supplementary Table 1. Search Queries**

**PubMed**

| **#** | **Term** |
| --- | --- |
| 1 | Breast Neoplasms[Mesh] OR "breast tumor"[TIAB] OR "breast tumors"[TIAB] OR "breast cancer"[TIAB] OR "breast cancers"[TIAB] OR "breast neoplasm"[TIAB] OR "breast neoplasms"[TIAB] |
| 2 | Cardiotoxicity[Mesh] OR "Cardiotoxicity"[TIAB] OR "Cardiotoxicities"[TIAB] OR "Cardiac Toxicity"[TIAB] OR "Cardiac Toxicities"[TIAB] OR "myocardial dysfunction"[TIAB] OR "heart failure"[TIAB] OR "coronary artery disease"[TIAB] OR "valvular disease"[TIAB] OR "arrythmia"[TIAB] OR "arterial hypertension"[TIAB] OR "pulmonary hypertension"[TIAB] OR "thromboembolic disease"[TIAB] OR "peripheral vascular disease"[TIAB] OR "stroke"[TIAB] OR "pericardial disease"[TIAB] OR "pleural effusion" OR "autonomic dysfunction"[TIAB] |
| 3 | "symptom"[TIAB] OR "Symptom Assessment"[Mesh] OR "symptom assessment"[TIAB] OR "symptom evaluation"[TIAB] OR "fatigue"[TIAB] OR "weakness"[TIAB] OR "lack of energy"[TIAB] OR "pain"[TIAB] OR "anorexia"[TIAB] OR "poor appetite"[TIAB] OR "cachexia"[TIAB] OR "vomit"[TIAB] OR "dry mouth"[TIAB] OR "diarrhea"[TIAB] OR "constipation"[TIAB] OR "loss of appetite"[TIAB] OR "insomnia"[TIAB] OR "sleep disturbance"[TIAB] OR "sleep difficulty"[TIAB] OR "breathless at night"[TIAB] OR "dyspnea"[TIAB] OR "exertional dyspnea"[TIAB] OR "DOE"[TIAB] OR "orthopnea"[TIAB] OR "shortness of breath"[TIAB] OR "edema"[TIAB] OR "swelling"[TIAB] OR "jugular vein distension"[TIAB] OR "weight gain"[TIAB] OR "weight loss"[TIAB] OR "bloat dizziness"[TIAB] OR "palpitation"[TIAB] OR "chest pain"[TIAB] OR "sweat"[TIAB] OR "anxiety"[TIAB] OR "cognitive dysfunction"[TIAB] OR "cough"[TIAB] OR "sexual dysfunction"[TIAB] OR "neuropathy"[TIAB] OR "dry mouth"[TIAB] OR "sexual problem"[TIAB] OR "numbness"[TIAB] OR "itching"[TIAB] |
| 4 | "Consolidation Chemotherapy"[Mesh] OR "Induction Chemotherapy"[Mesh] OR "Maintenance Chemotherapy"[Mesh] OR "Chemotherapy, Adjuvant"[Mesh] OR "consolidation chemotherapy"[TIAB] OR "consolidation chemotherapies"[TIAB] OR "induction chemotherapy"[TIAB] OR "induction chemotherapies"[TIAB] OR "maintenance chemotherapy"[TIAB] OR "maintenance chemotherapies"[TIAB] OR "adjuvant chemotherapy"[TIAB] OR "adjuvant chemotherapies"[TIAB] OR "chemotherapy"[TIAB] OR "chemotherapies"[TIAB] |
| 5 | #1 AND #2 AND #3 AND #4 |
| 6 | In the result of #5, we utilized the PubMed filter function to narrow down the results to English literature published after 2010. |

**Embase**

| **#** | **Term** |
| --- | --- |
| **1** | breast tumor'/exp OR 'breast tumor':ti,ab OR 'breast tumors':ti,ab OR 'breast cancer':ti,ab OR 'breast cancers':ti,ab OR 'breast neoplasm':ti,ab OR 'breast neoplasms':ti,ab |
| **2** | cardiotoxicity'/exp OR 'Cardiotoxicities':ti,ab OR 'Cardiac Toxicity':ti,ab OR 'Cardiac Toxicities':ti,ab OR 'myocardial dysfunction':ti,ab OR 'heart failure':ti,ab OR 'coronary artery disease':ti,ab OR 'valvular disease':ti,ab OR 'arrthymia':ti,ab OR 'arterial hypertension':ti,ab OR 'pulmonary hypertension':ti,ab OR 'thromboembolic disease':ti,ab OR 'peripheral vascular disease':ti,ab OR 'stroke':ti,ab OR 'pericardial disease':ti,ab OR 'pleural effusion' OR 'autonomic dysfunction':ti,ab |
| **3** | symptom'/exp OR 'symptom':ti,ab OR 'symptom assessment'/exp OR 'symptom assessment':ti,ab OR 'symptom evaluation':ti,ab OR 'fatigue':ti,ab OR 'weakness':ti,ab OR 'lack of energy':ti,ab OR 'pain':ti,ab OR 'anorexia':ti,ab OR 'poor appetite':ti,ab OR 'cachexia':ti,ab OR 'vomit':ti,ab OR 'dry mouth':ti,ab OR 'diarrhea':ti,ab OR 'constipation':ti,ab OR 'loss of appetite':ti,ab OR 'insomnia':ti,ab OR 'sleep disturbance':ti,ab OR 'sleep difficulty':ti,ab OR 'breathless at night':ti,ab OR 'dyspnea':ti,ab OR 'exertional dyspnea':ti,ab OR 'DOE':ti,ab OR 'orthopnea':ti,ab OR 'shortness of breath':ti,ab OR 'edema':ti,ab OR 'swelling':ti,ab OR 'jugular vein distension':ti,ab OR 'weight gain':ti,ab OR 'weight loss':ti,ab OR 'bloat dizziness':ti,ab OR 'palpitation':ti,ab OR 'chest pain':ti,ab OR 'sweat':ti,ab OR 'anxiety':ti,ab OR 'cognitive dysfunction':ti,ab OR 'cough':ti,ab OR 'sexual dysfunction':ti,ab OR 'neuropathy':ti,ab OR 'dry mouth':ti,ab OR 'sexual problem':ti,ab OR 'numbness':ti,ab OR 'itching':ti,ab |
| 4 | chemotherapy'/exp OR 'chemotherapy':ti,ab OR 'consolidation chemotherapy':ti,ab OR 'consolidation chemotherapies':ti,ab OR 'induction chemotherapy':ti,ab OR 'induction chemotherapies':ti,ab OR 'maintenanace chemotherapy':ti,ab OR 'maintenance chemotherapies':ti,ab OR 'adjuvant chemotherapy':ti,ab OR 'adjuvant chemotherapies':ti,ab |
| 6 | #1 AND #2 AND #3 AND #4 |
| 7 | #1 AND #2 AND #3 AND #4 AND [english]/lim AND [2010-2023]/py |

**CINAHL**

| **#** | **Term** |
| --- | --- |
| **1** | (MH "Breast Neoplasms+") |
| **2** | TI("breast tumor" OR "breast tumors" OR "breast cancer" OR "breast cancers" OR "breast neoplasm" OR "breast neoplasms") |
| **3** | AB("breast tumor" OR "breast tumors" OR "breast cancer" OR "breast cancers" OR "breast neoplasm" OR "breast neoplasms") |
| **4** | S1 OR S2 OR S3 |
| **5** | (MH "Cardiotoxicity") |
| **6** | TI("Cardiotoxicities" OR "Cardiac Toxicity" OR "Cardiac Toxicities" OR "myocardial dysfunction" OR "heart failure" OR "coronary artery disease" OR "valvular disease" OR "arrthymia" OR "arterial hypertension" OR "pulmonary hypertension" OR "thromboembolic disease" OR "peripheral vascular disease" OR "stroke" OR "pericardial disease" OR "pleural effusion" OR "autonomic dysfunction") |
| **7** | AB("Cardiotoxicities" OR "Cardiac Toxicity" OR "Cardiac Toxicities" OR "myocardial dysfunction" OR "heart failure" OR "coronary artery disease" OR "valvular disease" OR "arrthymia" OR "arterial hypertension" OR "pulmonary hypertension" OR "thromboembolic disease" OR "peripheral vascular disease" OR "stroke" OR "pericardial disease" OR "pleural effusion" OR "autonomic dysfunction") |
| **8** | S5 OR S6 OR S7 |
| **9** | (MH "Symptoms+") OR (MH "Signs and Symptoms+") |
| **10** | TI("symptom" OR "symptom assessment" OR "symptom evaluation" OR "fatigue" OR "weakness" OR "lack of energy" OR "pain" OR "anorexia" OR "poor appetite" OR "cachexia" OR "vomit" OR "dry mouth" OR "diarrhea" OR "constipation" OR "loss of appetite" OR "insomnia" OR "sleep disturbance" OR "sleep difficulty" OR "breathless at night" OR "dyspnea" OR "exertional dyspnea" OR "DOE" OR "orthopnea" OR "shortness of breath" OR "edema" OR "swelling" OR "jugular vein distension" OR "weight gain" OR "weight loss" OR "bloat dizziness" OR "palpitation" OR "chest pain" OR "sweat" OR "anxiety" OR "cognitive dysfunction" OR "cough" OR "sexual dysfunction" OR "neuropathy" OR "dry mouth" OR "sexual problem" OR "numbness" OR "itching") |
| **11** | AB("symptom" OR "symptom assessment" OR "symptom evaluation" OR "fatigue" OR "weakness" OR "lack of energy" OR "pain" OR "anorexia" OR "poor appetite" OR "cachexia" OR "vomit" OR "dry mouth" OR "diarrhea" OR "constipation" OR "loss of appetite" OR "insomnia" OR "sleep disturbance" OR "sleep difficulty" OR "breathless at night" OR "dyspnea" OR "exertional dyspnea" OR "DOE" OR "orthopnea" OR "shortness of breath" OR "edema" OR "swelling" OR "jugular vein distension" OR "weight gain" OR "weight loss" OR "bloat dizziness" OR "palpitation" OR "chest pain" OR "sweat" OR "anxiety" OR "cognitive dysfunction" OR "cough" OR "sexual dysfunction" OR "neuropathy" OR "dry mouth" OR "sexual problem" OR "numbness" OR "itching") |
| **12** | S9 OR S10 OR S11 |
| **13** | (MH "Induction Chemotherapy") OR (MH "Consolidation Chemotherapy") OR (MH "Chemotherapy, Adjuvant+") OR (MH "Chemotherapy, Cancer+") |
| **14** | TI("chemotherapy" OR "chemotherapies" OR "consolidation chemotherapy" OR "consolidation chemotherapies" OR "induction chemotherapy" OR "induction chemotherapies" OR "maintenance chemotherapy" OR "maintenance chemotherapies" OR "adjuvant chemotherapy" OR "adjuvant chemotherapies") |
| **15** | AB("chemotherapy" OR "chemotherapies" OR "consolidation chemotherapy" OR "consolidation chemotherapies" OR "induction chemotherapy" OR "induction chemotherapies" OR "maintenance chemotherapy" OR "maintenance chemotherapies" OR "adjuvant chemotherapy" OR "adjuvant chemotherapies") |
| **16** | S13 OR S14 OR S15 |
| **17** | S4 AND S8 AND S12 AND S16 |
| **18** | In the result of #5, we utilized the CINAHL filter functions to narrow down the results to English literature published after 2010 |
